# An Optimized Marinopyrrole A Derivative Targets 6-Phosphoglucosamine Synthetase to Inhibit Methicillin-Resistant *Staphylococcus aureus*

**DOI:** 10.1021/acscentsci.4c01167

**Published:** 2024-10-25

**Authors:** Fusheng Guo, Fan Xiao, Hao Song, Xiaoyong Li, Yaxin Xiao, Yong Qin, Xiaoguang Lei

**Affiliations:** † Beijing National Laboratory for Molecular Sciences, Key Laboratory of Bioorganic Chemistry and Molecular Engineering of Ministry of Education, College of Chemistry and Molecular Engineering, Peking University, Beijing 100871, China; ‡ Key Laboratory of Drug Targeting and Drug Delivery Systems of the Ministry of Education, Department of Medicinal Natural Products, West China School of Pharmacy, Sichuan University, Chengdu 610041, China; § Peking-Tsinghua Center for Life Science, Academy for Advanced Interdisciplinary Studies, Peking University, Beijing 100871, China; ∥ Institute for Cancer Research, Shenzhen Bay Laboratory, Shenzhen 518107, China

## Abstract

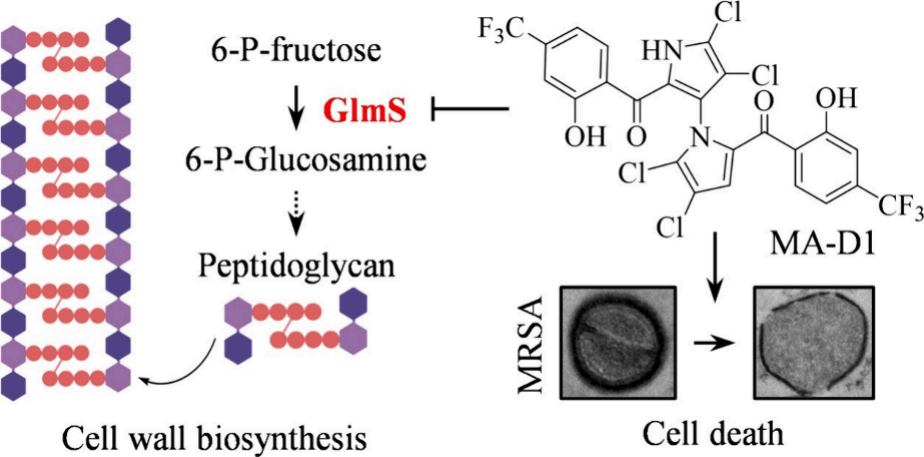

Methicillin-resistant *Staphylococcus aureus* (MRSA) is a common pathogenic bacterium that causes clinical infection and has become one of the most prominent antibiotic-resistant bacteria in the world. There is a pressing need to develop new antibiotics based on novel modes of action to combat increasingly severe MRSA infection. Marinopyrrole A (MA), a natural product extracted from marine *Streptomyces* in 2008, has a unique bipyrrole chemical skeleton and shows potent antibacterial activity against MRSA. However, its mode of action is still elusive. Herein, we developed an optimized MA derivative, MA-D1, and applied a chemoproteomic approach to reveal that MA-D1 performs its anti-MRSA activity by directly targeting 6-phosphoglucosamine synthetase (GlmS) to cause the breakdown of bacterial cell wall biosynthesis. Computational and experimental studies showed that MA-D1 interacts with the key R381 and E382 residues of GlmS in a novel binding pocket. Furthermore, MA-D1 showed a low resistance frequency for MRSA treatment and was also sensitive against the linezolid-, vancomycin-, or teicoplanin-resistant MRSA strains. MA-D1 also showed *in vivo* antibiotic efficacy in multiple animal models. This study demonstrates the promising potential of targeting GlmS to develop a new class of antibiotics to control MRSA pathogen infection.

## Abstract 2

Marinopyrrole A derivative MA-D1 shows anti-MRSA activity by targeting GlmS to inhibit cell wall biosynthesis. This discovery provides a novel target and a lead for developing new antibiotics.

## Introduction

Antibiotic resistance poses a significant risk to public health; for example, methicillin-resistant *Staphylococcus aureus* (MRSA) is a common pathogenic bacterium that causes clinical infections and has become a leading pathogen in hospital infections worldwide.^1^ Vancomycin, daptomycin, and linezolid are considered the last resort for treating MRSA infection, but worryingly, the corresponding drug-resistant bacteria have emerged in recent years.^2−4^ Therefore, there is an urgent need for the development of new antibiotics with novel antibacterial mechanisms to overcome antibiotic resistance problems.

**Scheme 1:**
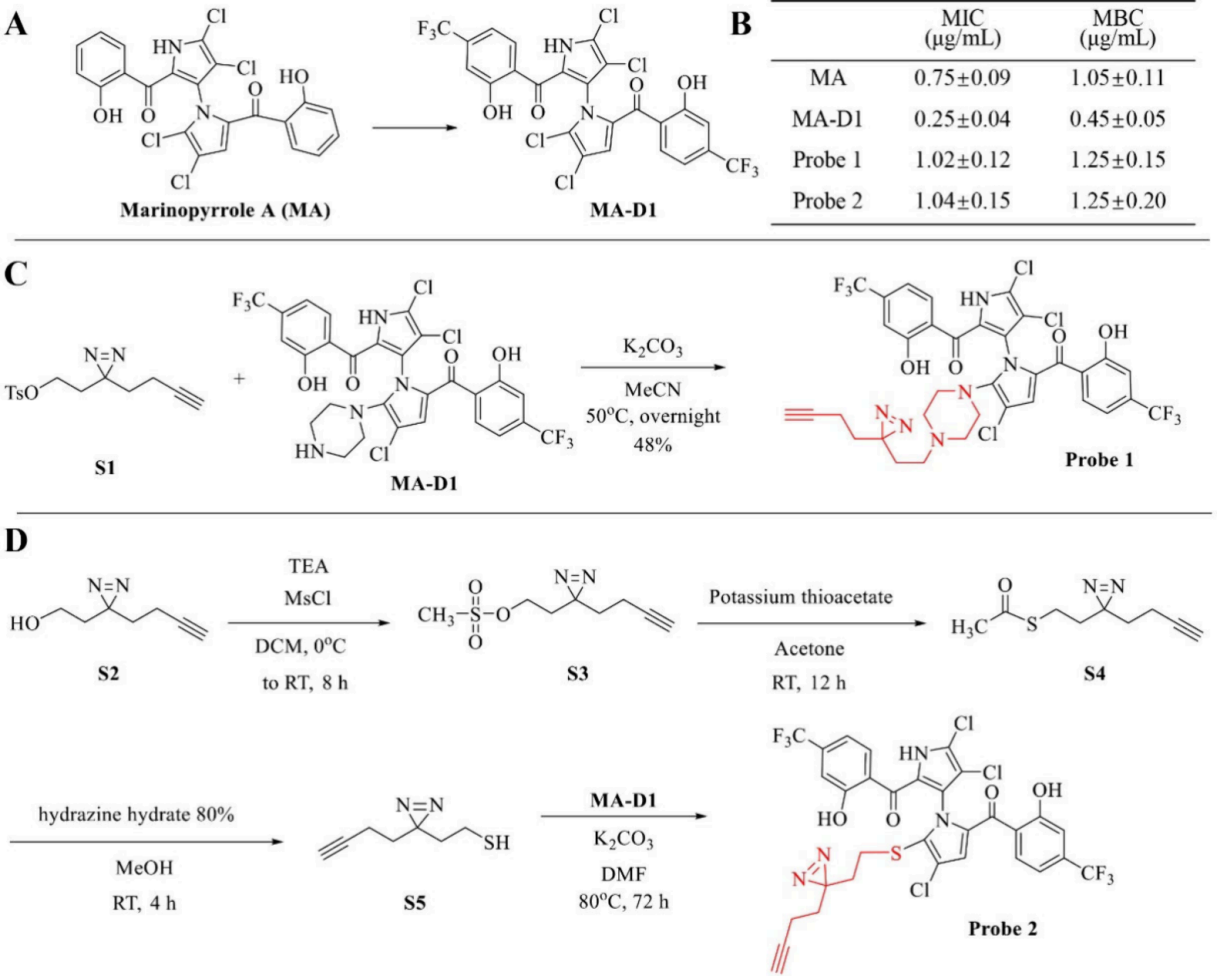
(A) MA-D1 Synthesis (Our Previous Work), (B) Antibacterial Activity (MIC and MBC) of Compounds against MRSA, and (C–D) Chemical Synthesis of Two Clickable and Photoreactive MA-D1 Probes for Biological Target Identification Values are the means ± SEM for three biological replicates.

Marinopyrrole A (MA), a natural product first isolated from *Streptomyces* strain CNQ-418 in a marine sediment sample in 2008, showed potent antitumor and anti-MRSA activities.^5^ MA has an uncommon bipyrrole chemical structure, different from all clinically used antibiotics. Considering its good antibacterial activity and unique chemical structure, the Qin group developed the total synthesis route of MA,^6^ and several groups synthesized a series of MA derivatives to increase the antibacterial activity.^7−10^ The best derivative, MA-D1 (Scheme 1A), had excellent antibacterial activity to MRSA and vancomycin-resistant *Enterococcus* (VRE), suggesting MA-D1 owns a potentially different mode of action from β-lactam and glycopeptide antibiotics.^8^ Mcl-1 and actin are two identified antitumor targets of MA in mammalian cells,^11,12^ but neither of these two proteins existed in bacteria. Collectively, since MA was discovered nearly 20 years ago, the antibacterial biological mechanisms of MA or its optimized derivative MA-D1 have not been elucidated.

Bacterial cell wall synthesis is essential for bacterial survival and growth, and some clinically used antibiotics induce bacteria death by targeting the cell wall biosynthesis pathway. For example, glycopeptides (vancomycin) and β-lactam (penicillin) antibiotics perform their antibacterial activities by targeting the transpeptidase in the peptidoglycan biosynthetic pathway.^13^ Peptidoglycan is the main component of bacterial cell walls, and the glycosyl donor of peptidoglycan biosynthesis is UDP-GlcNAc, the final product of the hexosamine biosynthesis pathway. 6-Phosphoglucosamine synthetase (GlmS) catalyzes the conversion of fructose-6-phosphate to glucosamine-6-phosphate, the rate-limiting step in the hexosamine biosynthesis pathway.^14^ In the meantime, *S. aureus* could not grow healthily after GlmS knockout.^15,16^ Therefore, small-molecule inhibitors targeting GlmS may be a promising solution for developing novel antibiotics. Although some reported compounds, including anticapsin,^17^ 6-diazo-5-oxo-norleucine,^18^ and *N*^3^-(4-metoxyfumaroyl)-(*S*)-2,3-diaminopropanoic acid (FMDP),^19,20^ could inhibit GlmS enzymatic activity *in vitro*, they all lack good drug-like properties due to either the poor selectivity or low anti-MRSA activity *in vivo*. To date, no GlmS-targeted antibiotics have been developed to treat bacterial infections. Therefore, GlmS-targeted antibiotics may potentially fill the human antibiotic arsenal.

In this work, GlmS is first identified as a direct protein target for MA-D1 through chemoproteomics. MA-D1 can potently inhibit the enzymatic activity of GlmS. The transmission electron microscope confirmed that MA-D1 treatment could cause MRSA cell membrane breakage and cell wall thinning, which is consistent with the biological activity of GlmS in facilitating the synthesis of UDP-GlcNAc and subsequent cell wall formation. Meanwhile, MA-D1 shows low resistance development for MRSA treatment and is sensitive against various antibiotic-resistant bacteria in clinical use. Notably, MA-D1 effectively lowers the bacterial load in multiple infection animal models. Collectively, this research identifies the antimicrobial target, GlmS, for an optimized natural product MA derivative and provides a promising lead for antibiotic drug discovery to address the global threat of MRSA.

## Results and Discussion

### Design and Synthesis of the Clickable and Photoreactive Probes

In our previous studies, the total synthesis route of natural product MA was developed^6^ and a series of MA derivatives were synthesized to increase the antibacterial activity.^8^ The derivative MA-D1, with a strong electron-withdrawing group (CF_3_) (Scheme 1A), exhibited the most potent antibacterial activity to MRSA (Scheme 1B). With a highly active compound in hand, we plan to investigate the biological targets of MA-D1 through chemical proteomics. We designed and prepared two structurally diverse probes derived from compound MA-D1, each containing an alkyne group and a photoreactive diazirine group. Probe 1 was directly prepared in 48% yield from MA-D1 with the minimalist linker S1 (Scheme 1C), and probe 2 was prepared in 19% yield from MA-D1 with the minimalist linker S5, which was derived from S2 through a substitution reaction and desulfurization ester reaction (Scheme 1D). The biological activity comparable with that of the parent compound is a necessary rule for a qualified probe to ensure the same binding targets as the parent compound. Probe 1 and probe 2 had good minimal inhibitory concentration (MIC) and minimum bactericidal concentration (MBC) values for MRSA treatment (Scheme 1B). Together, probes 1 and 2 can be advanced into chemical proteomics to search for the biological binding targets of MA-D1.

### Proteomic Profiling of Proteins Potentially Interacting with MA-D1

**Figure 1:**
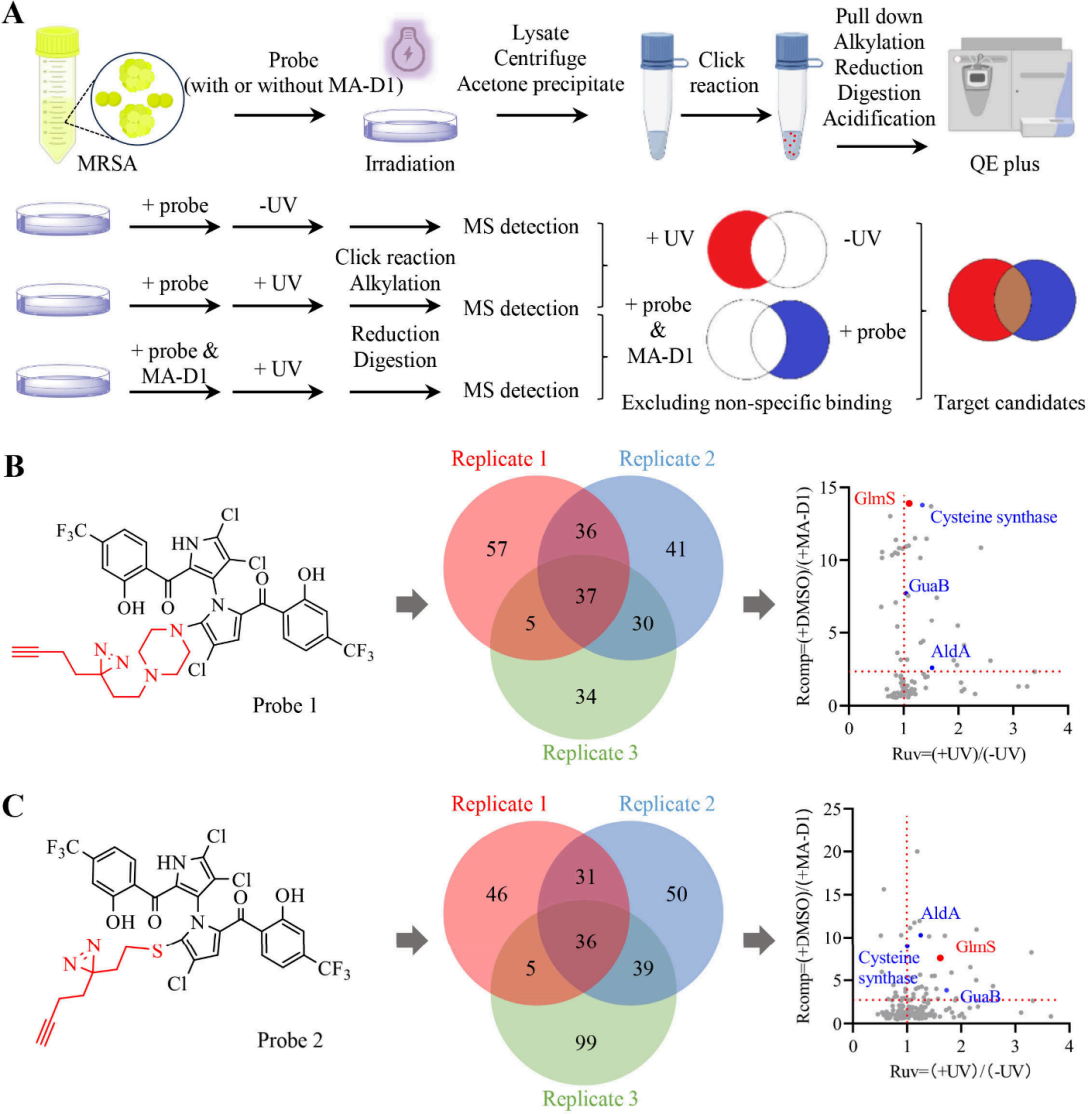
MS-based profiling of MA-D1-interacting proteins in living MRSA. (A) MRSA cells were incubated with either probe alone or with probe and MA-D1 then labeled with UV treatment, followed by click reaction with *N*_3_-biotin, pull-down with streptavidin–agarose beads, alkylation, reduction, digestion, acidification, and QE plus detection. Three biological replicates were performed for each probe. (B) Probe 1 chemical structure (left), overlap of identified proteins by probe 1 in three biological replicates (middle), and volcano plot of protein candidates (right). (C) Probe 2 chemical structure (left), overlap of identified proteins by probe 2 in three biological replicates (middle), and volcano plot of protein candidates (right).

To efficiently conduct the proteomic profiling, we first performed gel-based profiling to explore the optimized labeling conditions in MRSA by in-gel fluorescence (Figure S1A). MRSA cells were treated with probe 1 or probe 2 of gradient concentration for 15 min and then irradiated with 365 nm UV light for another 15 min. In the meantime, probes and gradient concentrations of native MA-D1 were utilized to compete with probe-interacting proteins. The cross-linked proteins were conjugated with azide-FITC by a click reaction and analyzed by SDS-PAGE. The in-gel fluorescence results revealed clear bands at a 150 μM concentration of probes, and the probe-interacting proteins could be competed off by native 300 μM MA-D1 (Figure S1B). After determining the optimal concentration of probes for target labeling and native MA-D1 for competition, we next performed MS-based proteomic profiling of probe-interacting proteins in MRSA (Figure 1A). An MA-D1-induced competitive ratio (*R*_comp_) of 2.5 and UV-induced ratio (*R*_uv_) of 1 were chosen as the cutoffs to exclude proteins that only had interaction with probes or nonspecifically bind to streptavidin–agarose beads, and those detected in two out of three replicates are considered positive candidates. Finally, a total of 108 and 111 MA-interacting protein candidates were identified by probe 1 (Figure 1B) and probe 2 (Figure 1C), and 20 and 32 protein candidates met the above-mentioned restriction conditions (*R*_comp_ > 2.5, *R*_uv_ > 1) (Figures 1B and 1C). Inosine 5′-monophosphate dehydrogenase (GuaB), aldehyde dehydrogenase (AldA), cysteine synthase, and 6-phosphoglucosamine synthetase (GlmS) were simultaneously captured by two probes (Figures 1B and 1C).

### GlmS Is a Direct Biological Target of MA-D1

**Figure 2:**
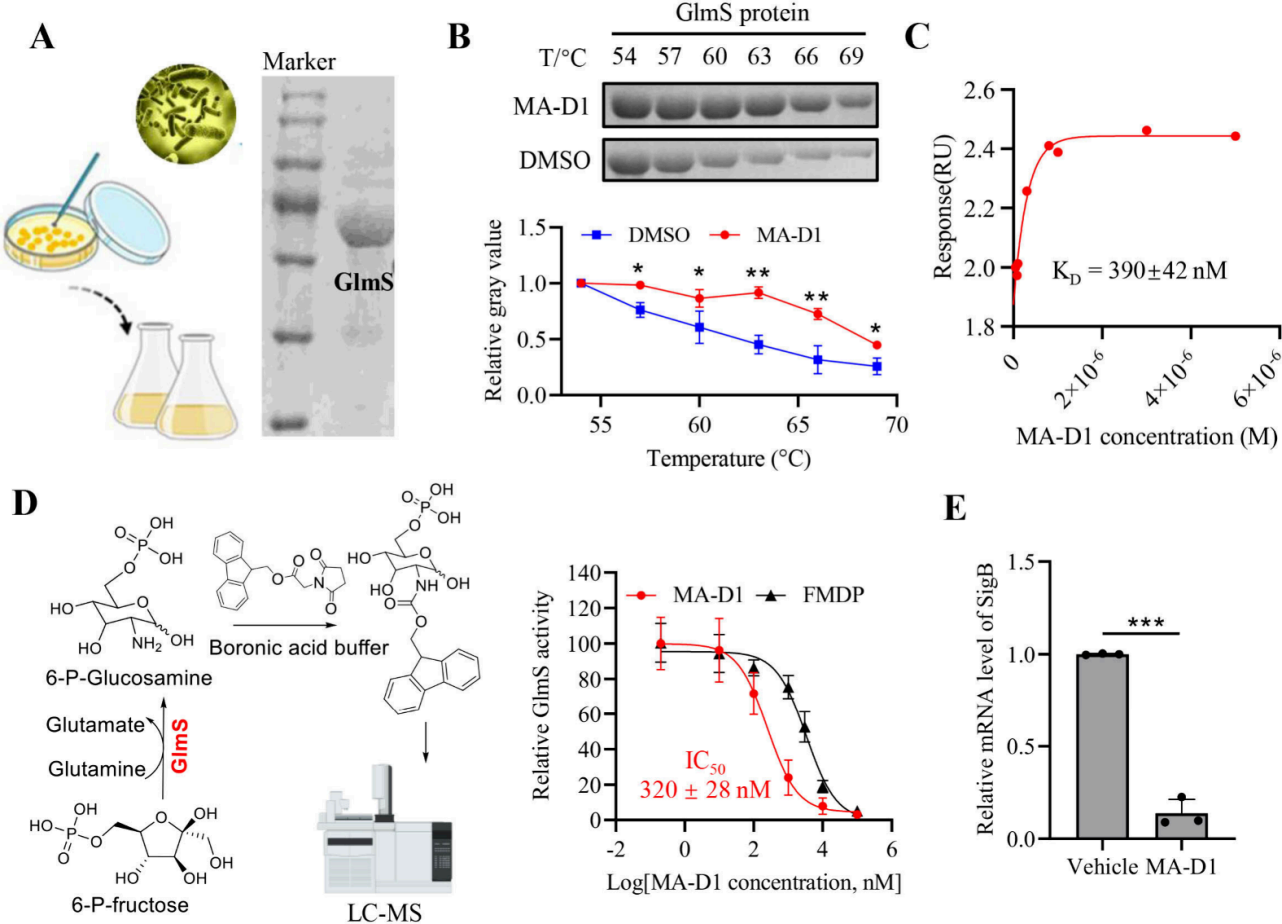
MA-D1 directly interacts with and inhibits the activity of GlmS protein. (A) GlmS protein was purified using the *E. coli* expression system. (B) The effect of MA-D1 on the thermal stability of GlmS protein (up) and the relative gray values was quantified (down). (C) The binding affinity between MA-D1 and purified GlmS protein based on SPR assay. (D) Enzyme activity detection assay was established based on the GlmS-catalyzed chemical reaction (left), and the dose–response curve of MA-D1 on inhibiting the GlmS enzymatic activity was measured (right). The reported FMDP was used as a positive control. (E) The relative mRNA level of SigB in the presence and absence of MA-D1 (0.5 μM) in MRSA. Values are the means ± SEM; *P < 0.05, **P < 0.01, and ***P < 0.001.

We purified the AldA, GuaB, and cysteine synthase proteins of *S. aureus*. We established their enzymatic detection assays, but MA-D1 showed little activity against these enzymes, suggesting that these candidates are not the biological targets of MA-D1 (Figures S2–S4). GlmS, an essential enzyme in the hexosamine biosynthetic pathway, catalyzes fructose-6-phosphate to glucosamine-6-phosphate in bacteria. We also purified the GlmS protein (Figure 2A), and the thermal stability of the GlmS protein could be increased by MA-D1 treatment, suggesting the direct interaction of MA-D1 with GlmS (Figure 2B). Next, the direct interaction between MA-D1 and GlmS was assessed by using a surface plasmon resonance (SPR) test, revealing an affinity (*K*_d_) of 390 nM (Figure 2C), confirming the direct binding of GlmS with MA-D1. Next, we established an *in vitro* enzymatic assay based on the GlmS-catalyzed chemical reaction to evaluate whether MA-D1 could inhibit the enzymatic activity of GlmS, and the results showed that MA-D1 could potently inhibit the GlmS-catalyzed enzymatic activity with an IC_50_ value of 320 nM (Figure 2D). In kinetic measurements, the presence of MA-D1 caused a reduced *V*_max_, but an unchanged *K*_m_ of GlmS, suggesting that MA-D1 is a noncompetitive inhibitor (Figure S5). In the meantime, global regulator sigma factor σB (SigB) is a reported downstream target gene of GlmS in MRSA;^21^ MA-D1 treatment indeed significantly inhibits the transcript of SigB (Figure 2E). Together, these data suggest that GlmS is a direct biological target of MA-D1.

### MA-D1 Induces MRSA Death in a GlmS-Dependent Manner

**Figure 3:**
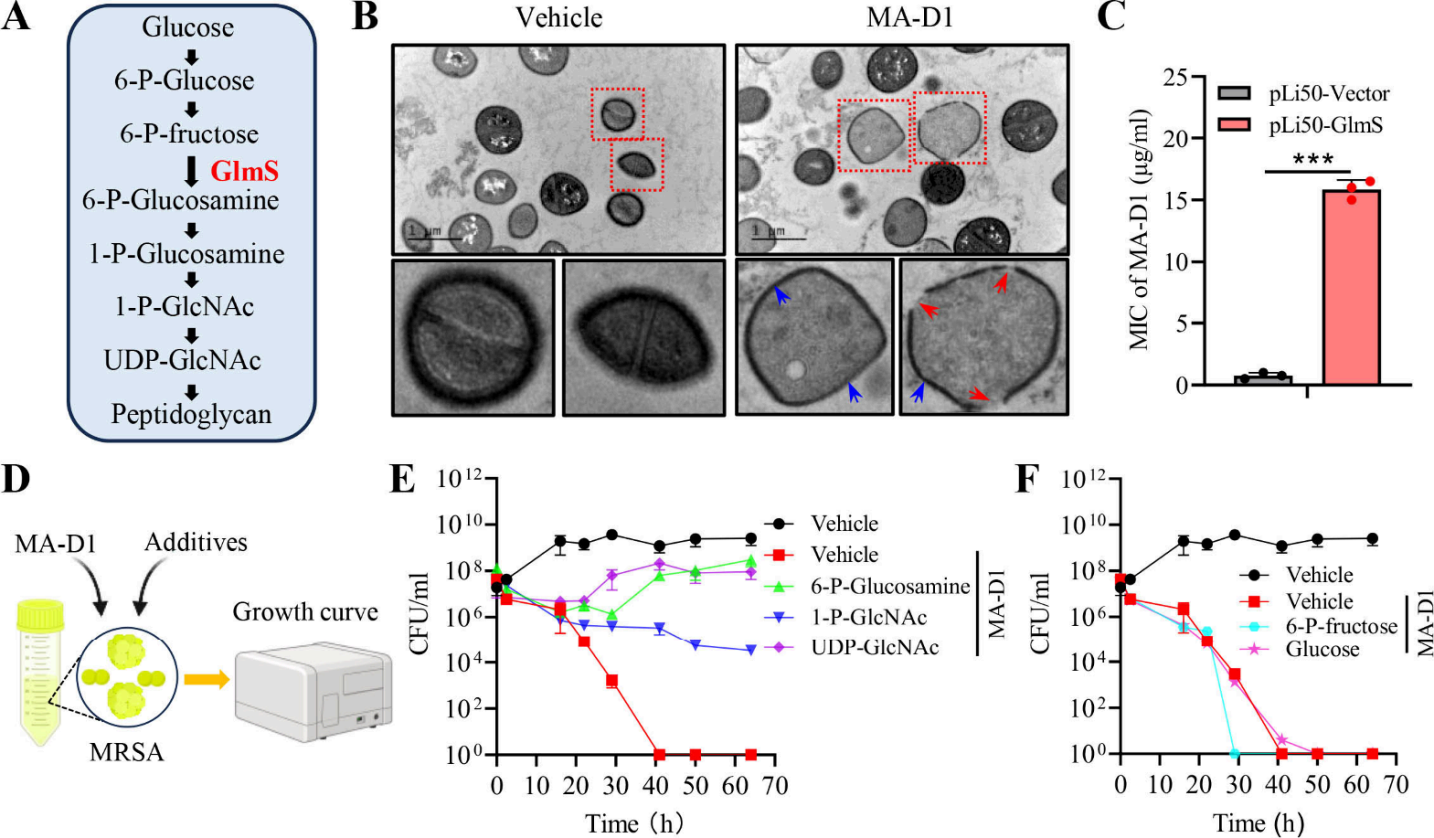
MA-D1 inhibits GlmS to block cell wall biosynthesis. (A) The GlmS-medicated enzymatic reaction regulates cell wall formation in bacteria. (B) Transmission electron microscopy was used to determine the morphological change of MRSA after MA-D1 (4 μg/mL) or vehicle treatment. Red arrows, the broken cell membrane; blue arrows, the thinning cell wall. (C) The MIC values of MA-D1 against MRSA transfected with pLi50-GlmS plasmid or empty vector. (D–F) The MRSA growth curve under MA-D1 (0.5 μg/mL) treatment with or without 1 mg/mL 6-P-glucosamine, 1-P-GlcNAc, UDP-GlcNAc, 6-P-fructose, or glucose, respectively. Values are the means ± SEM; ***P < 0.001.

In bacteria, GlmS catalyzes the conversion of fructose-6-phosphate to glucosamine-6-phosphate to regulate the production of UDP-GlcNAc. UDP-GlcNAc is the major glycosyl donor of peptidoglycan, one of the main components of bacteria cell walls (Figure 3A). We surmise that MA-D1 may perform its anti-MRSA activity by inhibiting the production of UDP-GlcNAc and inducing subsequent cell wall collapse by targeting GlmS. Indeed, the transmission electron microscope confirmed that MA-D1 treatment could cause MRSA cell membrane breakage and cell wall thinning (Figure 3B). The overexpression of GlmS could weaken the anti-MRSA activity of MA-D1 (Figure 3C). Next, we tested whether the different intermediates in the hexosamine biosynthetic pathway could rescue MA-D1-induced MRSA death (Figure 3D). MRSA could survive and grow under the pressure of MA-D1 in the presence of downstream substances of GlmS, including 6-P-glucosamine, 1-P-GlcNAc, and UDP-GlcNAc (Figure 3E), and the MIC and MBC of MA-D1 against MRSA increased significantly (Figure S6). In contrast, glucose and 6-P-fructose are the upstream substances of GlmS in the hexosamine biosynthetic pathway; their supplementation failed to rescue MRSA from the MA-D1-induced death (Figure 3F), and MA-D1 also kept the MIC and MBC values against MRSA (Figure S6). Together, these results suggest that MA-D1 has antibacterial activity in a GlmS-dependent manner.

### Binding Mechanism and Functional Correlation between MA-D1 and GlmS

After exploring the inhibitory effect of MA-D1 on the pharmacological function of GlmS, we planned to further explore the molecular mechanism of MA-D1 as a GlmS inhibitor. Several key factors have been reported to inhibit the GlmS enzymatic activity. First, glutamine analogues can compete with glutamine to inhibit GlmS activity.^16^ Second, GlmS has two oligomeric forms in the physiological state; the hexamer is inactive, but the dimeric form is active, so the balance of different GlmS protein forms is crucial for its enzymatic activity.^22^ Third, glutamine fructose-6-phosphate amidotransferase (GFAT), the human homologous protein of bacterial GlmS, can be inhibited by the negative feedback regulation of UDP-GlcNAc, which keeps the dynamic balance of products in the hexosamine biosynthetic pathway.^23^ Next, we separately verified the potential molecular mechanism of the MA-D1 inhibitory effect on GlmS.

**Figure 4:**
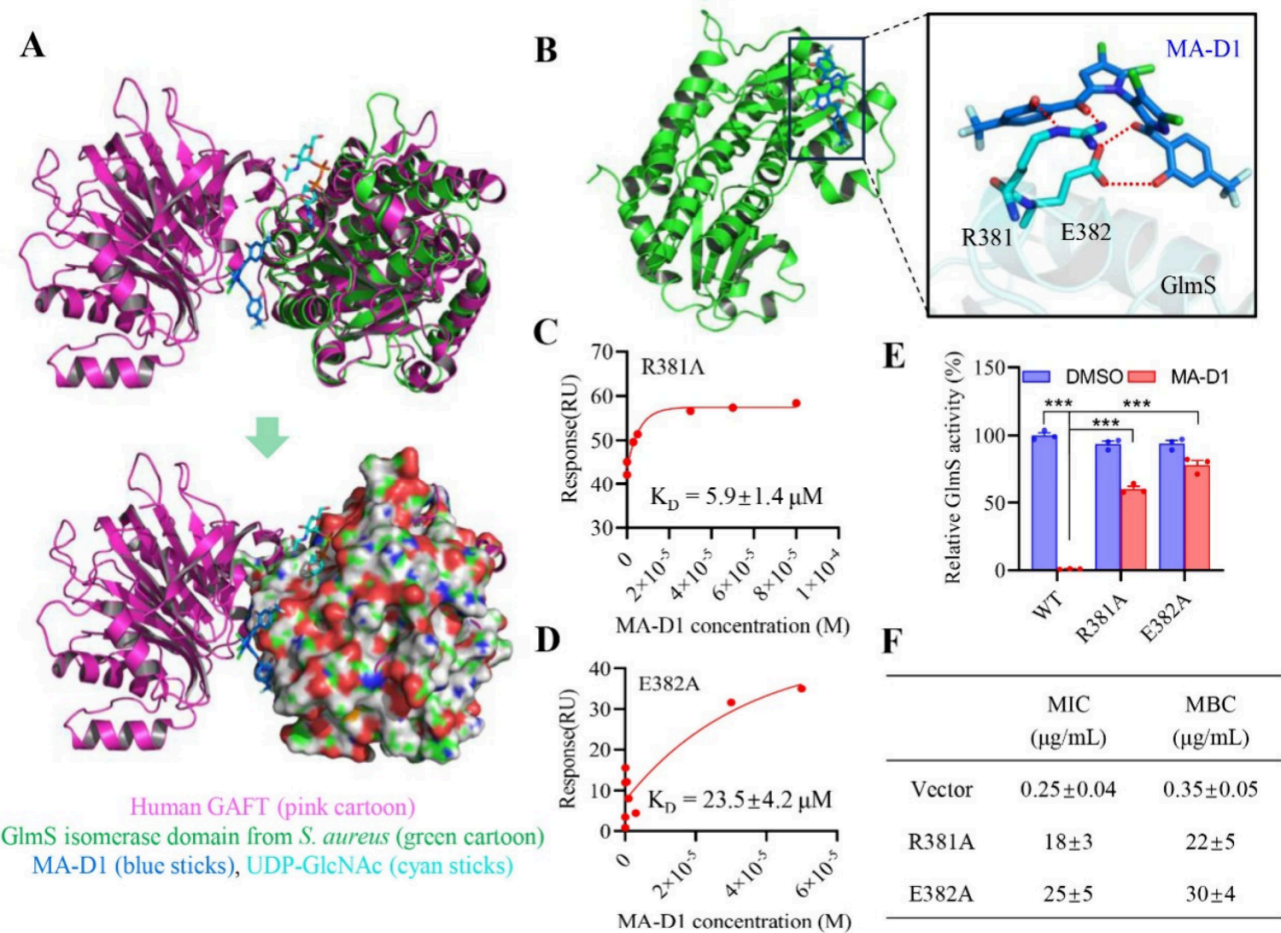
Molecular docking and functional correlation of MA-D1 binding with GlmS. (A) The molecular docking structure of MA-D1 binding on the interface of the GlmS isomerase domain is based on the reported GlmS structure from *S. aureus* (PDB 4S1W) using MOE software. In the meantime, the alignment between the docked MA-D1/GlmS isomerase domain complex and the reported UDP-GlcNAc/GFAT complex (PDB 6SVP) was conducted using PyMol software. (B) The key interaction between MA-D1 and GlmS isomerase domain. The hydrogen bonds between MA-D1 and key residues are indicated with dashed red lines. (C and D) The binding affinity between MA-D1 and GlmS protein with indicated key residue (R381A and E382A) mutations based on SPR assays. (E) The relative enzymatic activities of GlmS protein with indicated key residue (R381A and E382A) mutations with or without the MA-D1 treatment. (F) The MIC and MBC values of MA-D1 against MRSA transfected with empty vector or pLi50-GlmS R381A or E382A inactive mutant plasmids. Values are the means ± SEM; ***P < 0.001.

First, the high concentration of glutamine (1 mM) or 6-phosphate-fructose (1 mM) could not markedly destroy the binding affinity between MA-D1 and GlmS protein, suggesting the inhibitory activity of MA-D1 against GlmS is not achieved by direct competition of the GlmS’s native substrates (Figure S7). Second, both the native PAGE and size exclusion chromatography confirmed that MA-D1 did not influence the oligomeric forms of GlmS protein, suggesting MA-D1 also did not exert its inhibitory activity through converting the oligomeric forms of GlmS (Figure S8). Next, we sought to solve the MA-D1/GlmS complex structure to understand the binding pattern better. Unfortunately, we have made plenty of attempts to resolve the cocrystal structure between MA-D1 and GlmS, but all failed. One of the key reasons is boldly speculated to be due to the dynamic conformational change of GlmS. Although the cocrystal was not available, with the previously reported X-ray crystal structures in hand, including the reported GlmS isomerase domain structure from *S. aureus* (PDB 4S1W) and the UDP-GlcNAc/GFAT complex (PDB 6SVP) structure, molecular docking was performed to better understand the binding pattern between MA-D1 and GlmS. Similar to UDP-GlcNAc, MA-D1 compatibly binds to the surface of the GlmS isomerase domain located between the glutaminase domain and isomerase domain (Figure 4A) and forms four hydrogen bond interactions with the R381 and E382 on GlmS (Figure 4B). Based on our computational data, the hydrogen bond interaction between MA-D1 and GlmS is enhanced by the strong electron-withdrawing nature of CF_3_ groups on the benzene ring, which may be an important reason for the activity enhancement of MA-D1 compared to that of the parent natural product MA. Next, we mutated these two residues to confirm if the predicted key residues of GlmS contribute to the binding affinity and inhibitory activity of MA-D1. The binding affinity decreased more than 15-fold after the R381A mutation (Figure 4C), and the E382A mutation caused a nearly 60-fold decrease in the binding affinity (Figure 4D). Consistently, the enzymatic activity of GlmS with R381A or E382A could not be markedly inhibited by MA-D1 compared to the wild-type GlmS protein (Figure 4E), and the MIC and MBC values of MA-D1 markedly increased against MRSA transfected with GlmS-R381A and GlmS-E382A (Figure 4F). We elaborated on the binding mechanism between MA-D1 and GlmS based on computational and experimental studies.

### Low Resistance Frequency of MA-D1 for MRSA Treatment

**Figure 5:**
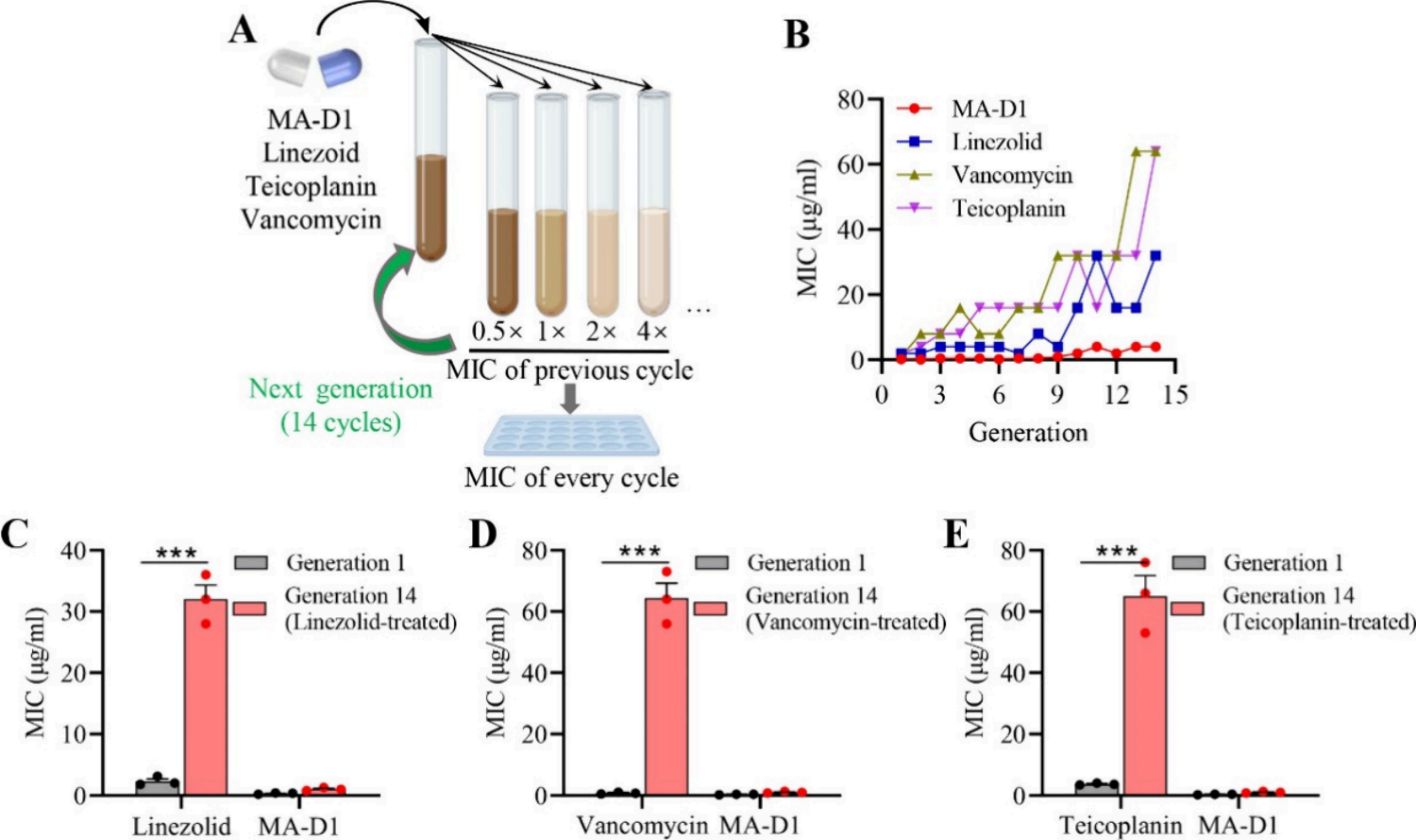
Low resistance development of MA-D1 for MRSA treatment. (A and B) Exposure of an antibiotic-sensitive strain of MRSA to sub-MIC dosing of MA-D1, linezolid, vancomycin, and teicoplanin for 14 sequential passages (A) and the MIC curves (B). The test was carried out three times independently with analogous results. (C–E) The sensitivity of MRSA to MA-D1 after 14 sequential cycles of treatment with sub-MIC dosing of linezolid (C), vancomycin (D), or teicoplanin (E). Values are the means ± SEM; ***P < 0.001.

Resistance development often determines the potential durability of an antibiotic. Further studies were conducted to evaluate the possibility that MRSA develops resistance to MA-D1. The drug resistance of MRSA to MA-D1, linezolid, vancomycin, or teicoplanin during serial passaging at the sub-MIC concentration was tested (Figure 5A). Vancomycin and teicoplanin resistance developed as early as passage 3, and the MIC value rose more than 30-fold after 14 generations; linezolid resistance developed as early as passage 8, and the MIC value also rose nearly 20-fold after 14 generations; but the anti-MRSA potency of MA-D1 was unaltered for the duration of the test (Figure 5B). More excitingly, MA-D1 showed powerful anti-MRSA potency against linezolid (Figure 5C), vancomycin (Figure 5D), or teicoplanin (Figure 5E) resistant MRSA after 14 passages. The results suggested that novel antibiotics targeting GlmS may provide superiority in preventing resistance development, and MA-D1 had the potential to combine with other antibiotics to treat resistant bacterial infections and reduce the frequency of drug-resistant mutation.

### MA-D1 Exhibits Potent Antibacterial Activity *In Vivo*

**Figure 6:**
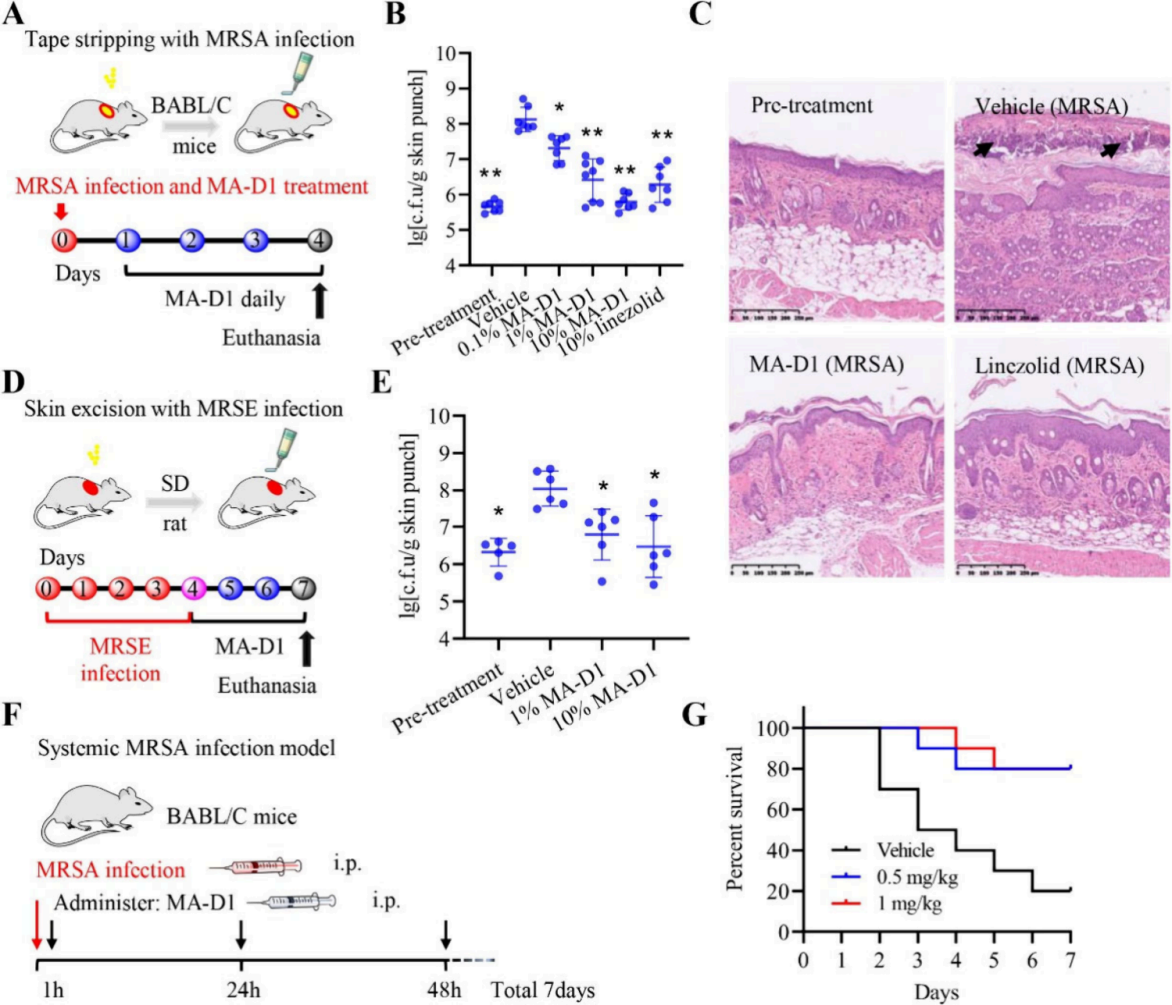
MA-D1 exhibits potent antibacterial activity in animals. (A–C) The efficacy of MA-D1 on alleviating MRSA infection in a mouse model (*n* = 6–7). The designed experimental pattern flowchart (A) and the c.f.u. of skin surface-attached bacteria in mice receiving MA-D1, linezolid, or vehicle following the skin infection with MRSA (B); the skin pathological sections were detected. Scale bar, 250 μm (C). (D and E) The efficacy evaluation of MA-D1 in a MRSE skin infection SD rat model (*n* = 5–6). The designed experimental pattern flowchart (D) and the c.f.u. of skin surface-attached bacteria in rats receiving MA-D1 or vehicle following the skin infection (E). (F and G) The efficacy of MA-D1 in a systemic MRSA infection mice model (*n* = 10). The designed experimental pattern flowchart (F) and the survival rate of mice receiving MA-D1 (0.5 and 1 mg/kg, i.p.) or vehicle following systemic infection with MRSA (G). Values are the means ± SEM; *P < 0.05 and **P < 0.01.

Given the novel mode of action and promising *in vitro* antibacterial activity of MA-D1, we next tested the toxicity, pharmacokinetic parameters, and efficacy of MA-D1 in alleviating MRSA infection. Cell culture safety profiling experiments showed that MA-D1 has moderate cytotoxicity toward mammalian cells (concentration causing 50% cell growth inhibition (IC_50_) > 3.5 μM, Figure S9A), and the hemolytic toxicity of MA-D1 was low in the effective anti-MRSA doses (0.25–2 μg/mL, Figure S9B). In mice, MA-D1 achieved a good half-life (6.3 h in i.v. and 8.2 h in p.o.), ideal maximum plasma concentration (3681 ng/mL in i.v. and 2004 ng/mL in p.o.), and acceptable oral bioavailability (11%) (Figure S9C). In the meantime, MA-D1 showed the highest level of exposure in the lung among multiple organs (Figure S9D), suggesting its therapeutic potential for lung infection. Next, we studied the efficacy of MA-D1 in murine infection animal models. First, the MRSA-infected skin topical animal model was established with BALB/C female mice (Figure 6A). MA-D1 topical application led to a robust reduction or even complete eradication of MRSA on the mice’s skin surface, verified by colony statistics (Figure 6B) and skin histological sections with H&E staining (Figure 6C). Then, we also tested the efficacy of MA-D1 for eradicating methicillin-resistant *Staphylococcus epidermidis* (MRSE) infection *in vivo* (Figure 6D). Topical MA-D1 treatment also could potently alleviate MRSE infection in rats (Figure 6E). Next, we studied the ability of MA-D1 to rescue mice from a lethal challenge of intraperitoneally administered MRSA (Figure 6F). Systemic administration of MA-D1 (0.5 and 1 mg/kg) markedly resulted in the rescue of infected mice (Figure 6G), and MA-D1 was hypotoxic at 1 mg/kg based on the organ-to-body weight ratio (Figure S9E) and hemolysis *in vivo* (Figure S9F). Finally, we also confirmed that the antibacterial activity of MA-D1 was reduced in mice with topical infection with MRSA expressing inactive GlmS-R381A or GlmS-E382A mutants (Figure S10). Together, these results illustrate that MA-D1 possesses a remarkable therapeutic effect in reducing infection against MRSA in animals.

## Conclusion

In summary, combining chemoproteomic, computational, and experimental studies, we first clarified that the optimized MA derivative MA-D1 performs potent anti-MRSA activity by directly targeting GlmS to induce the breakdown of bacteria cell wall biosynthesis. More importantly, no drugs targeting GlmS have been approved in the clinic, and highly druggable lead compounds are also lacking, so targeting GlmS for novel antibiotic development is an entirely novel mode of action that differs from all current antibiotics. In conclusion, this work not only elucidates the antibacterial mechanism of MA-D1 for the first time but also provides a novel drug target for developing novel antibiotics. Considering the novelty of the MA-D1 chemical skeleton, the low resistance frequency, and the novel anti-MRSA mechanism, it is promising to potentially derive an antibiotic with extended clinical durability based on the newly established relationships among MA-D1, GlmS, and potent anti-MRSA activity in this work.

## Supporting Information Available

General information, supplementary figures and tables, materials and methods of biological experiments, synthesis and characterization data, copies of ^1^H and ^13^C NMR spectra and HRMS, and references (PDF)
