# Supplementary material for: An Optimized Marinopyrrole A Derivative Targets 6-Phosphoglucosamine Synthetase to Inhibit Methicillin-Resistant Staphylococcus aureus
Source: ACS Cent Sci. 2024 Oct 25;10(11):2090–8. doi: 10.1021/acscentsci.4c01167 (PMC11613329; doi:10.1021/acscentsci.4c01167)
Supplement: Supplementary file 1 — oc4c01167_si_001.pdf [file oc4c01167_si_001.pdf]

Supporting Information

An optimized marinopyrrole A derivative targets 6-phosphoglucosamine synthetase to inhibit Methicillin-resistant *Staphylococcus aureus*

Fusheng Guo[1,3 †], Fan Xiao[1 †], Hao Song[2 †], Xiaoyong Li[2], Yaxin Xiao[2], Yong Qin[2] * and Xiaoguang Lei[1,3,4] *

[1] Beijing National Laboratory for Molecular Sciences, Key Laboratory of Bioorganic Chemistry and Molecular Engineering of Ministry of Education, College of Chemistry and Molecular Engineering, Peking University, Beijing 100871, China.

[2] Key Laboratory of Drug Targeting and Drug Delivery Systems of the Ministry of Education and State Key Laboratory of Biotherapy, Department of Medicinal Natural Products, West China School of Pharmacy, Sichuan University, Chengdu 610041, China.

[3] Peking-Tsinghua Center for Life Science, Academy for Advanced Interdisciplinary Studies, Peking University, Beijing 100871, China.

[4] Institute for Cancer Research, Shenzhen Bay Laboratory, Shenzhen 518107, China.

[†] These authors contributed equally to this work.

*Corresponding authors: yongqin@scu.edu.cn (Y. Q.); xglei@pku.edu.cn (X. L.).

Table of Contents

**I) General Information**	S3
**II) Supplementary figures and tables**	S4–S8
**III) Materials and methods of biology experiments**	S9-S15
**IV) Synthesis of probes**	S16–S17
**V) NMR Spectra**	S18-S22
**VI) Reference**	S23

I) General Information

Reactions required for anhydrous conditions were performed in flame-dried glassware under argon atmosphere and all reagents were purchased from commercial suppliers. Reactions were monitored by thin layer chromatography (TLC). The synthetic products were purified by column chromatography on silica gel (200–300 meshes) or by preparative LC, and determined by NMR spectrometer. ^{1}H NMR and ^{13}C NMR spectra were recorded on Varian INOVA-400/54 and Agilent DD2-600/54 spectrometer, in $CDCl_3$ solvent (reference peaks: ^{1}H NMR: 7.26 ppm; ^{13}C NMR: 77.16 ppm). High-resolution mass spectra (HRMS) were recorded on Bruker Apex IV FTMS or Agilent LC-MSD TOF ESI mass spectrometers.

Abbreviations:

DCM: dichloromethane

TEA: triethylamine

MsCl: methanesulfonyl chloride

PE: petroleum ether

EA: ethyl acetate

MeOH: methanol

$NaHCO_3$: sodium bicarbonate

K_2CO_3: potassium carbonate

DMF: *N,N*-dimethylformamide

TLC: thin layer chromatography

RT: room temperature

min: minutes

h: hours

II) Supplementary figures and tables

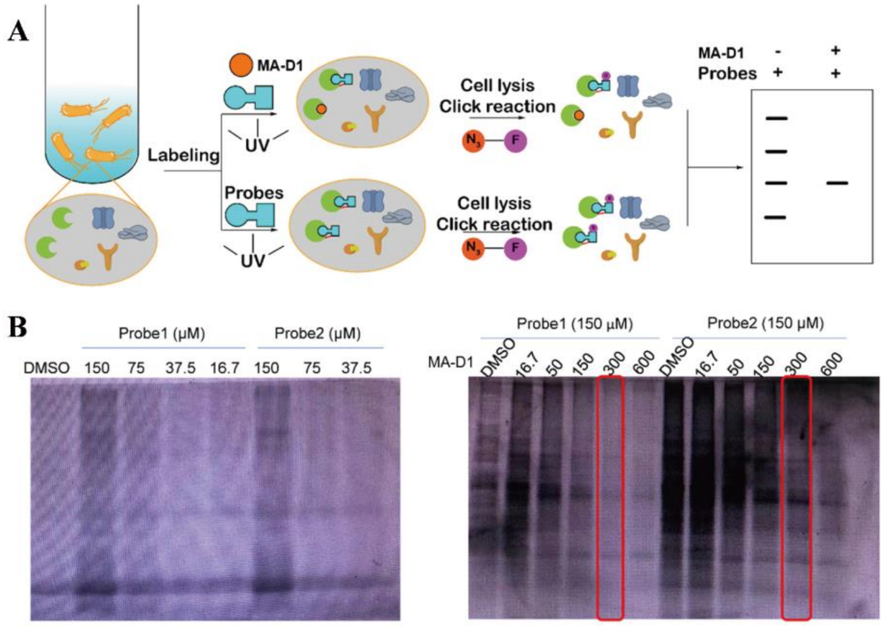

Figure S1. Gel-based profiling of MA-D1-interacting proteins in living cells. (**A**) Workflow for gel-based profiling of MA-D1-interacting proteins in living *S.aureus* cells. (**B**) Evaluation of labeling efficiency of two MA-D1 probes in living *S.aureus* cells by in-gel fluorescence.

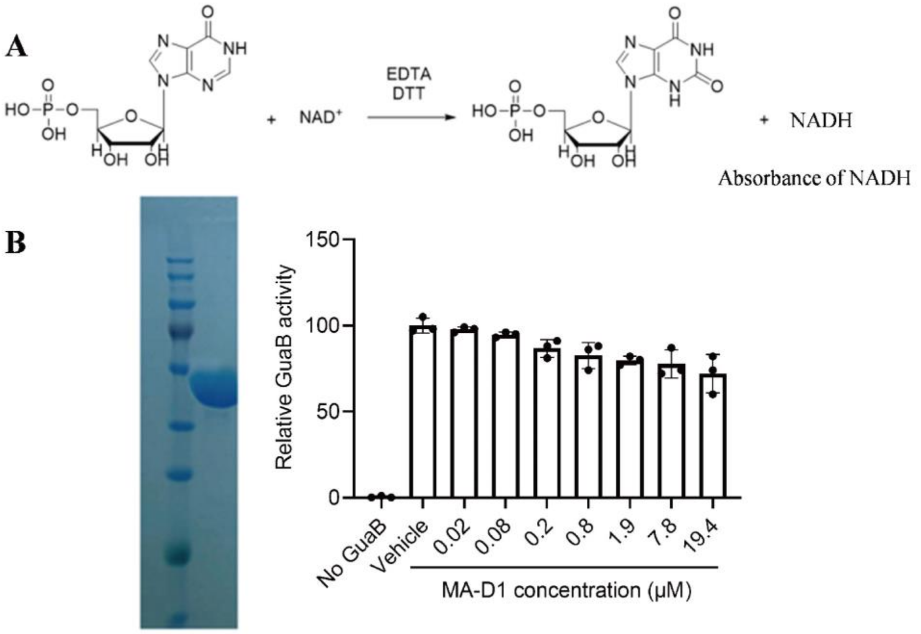

Figure S2. Verifying whether MA-D1 has the inhibitory activity of GuaB enzyme. (**A**) Enzyme activity detection assay based on the GuaB enzyme catalyzed chemical reaction. (**B**) GuaB protein was purified in *E.coli* expression system, then the enzymatic activity was verified at gradient concentration of MA-D1 . Gradient MA-D1 were pre-incubated with GuaB, then NAD^+, EDTA, DTT and IMP were added to trigger the enzymatic action at 25 °C for 24 hours, finally, the production of NADH was identified through monitoring the absorption at 340 nm.

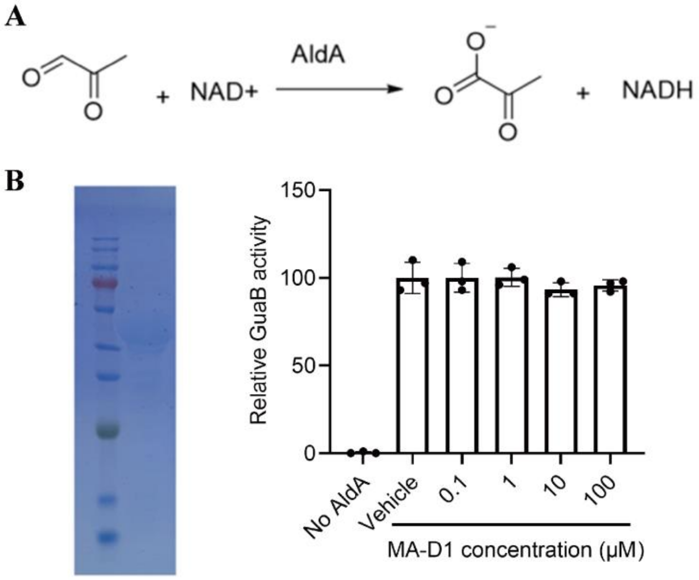

Figure S3. Verifying whether MA-D1 has the inhibitory activity of AldA enzyme. (**A**) Enzyme activity detection assay based on the AldA enzyme catalyzed chemical reaction. (**B**) AldA protein was purified in *E. coli* expression system, then the enzymatic activity was verified at gradient concentration of MA-D1. Gradient MA-D1 were pre-incubated with AldA for 4 hours, then NAD^+ and methylglyoxal were added to trigger the enzymatic action at 25℃, finally, the production of NADH was identified through monitoring the absorption at 340 nm.

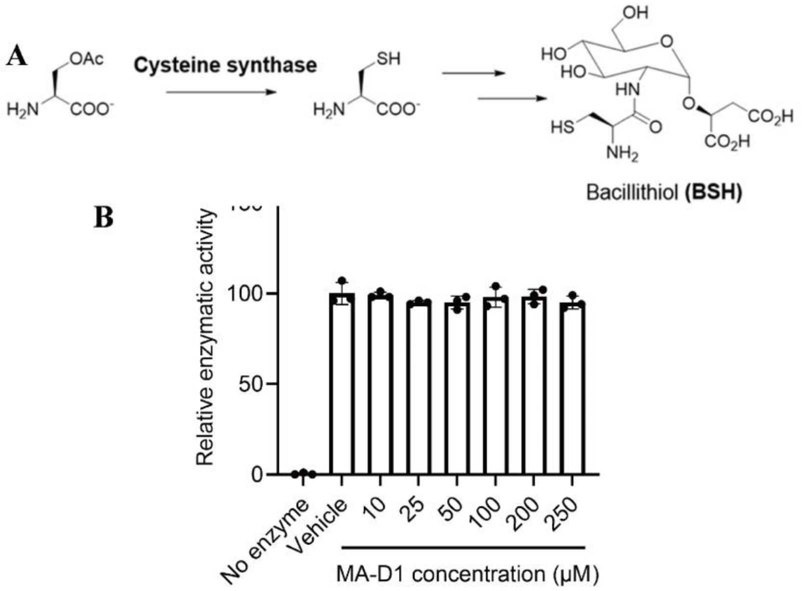

Figure S4. Verifying whether MA-D1 has the inhibitory activity of cysteine synthase. (**A**) Enzyme activity detection assay based on the cysteine synthase catalyzed chemical reaction. (**B**) Cysteine synthase protein was purified in the *E. coli* expression system, then the enzymatic activity was verified at gradient concentration of MA-D1. Gradient MA-D1 was pre-incubated with cysteine synthase for 4 hours, then derivative cysteine based on Ninhydrin method was detected by 560 nm characteristic absorption.

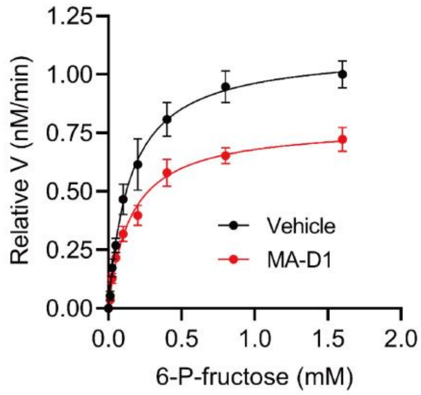

	Vehicle	MA-D1
K_m (mM)	0.15±0.02	0.16±0.03
V_{max}(nM/s)	1.11±0.05	0.78±0.04

Figure S5. The kinetic measurements of GlmS in the absence and presence of MA-D1 (0.1μM). Values are the means ± SEM, n =3.

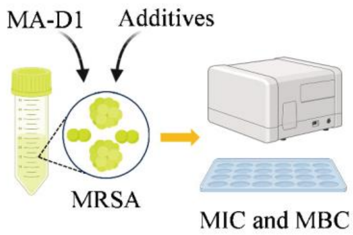

	MIC (μg/mL)	MBC (μg/mL)
Vehicle	0.25±0.04	0.35±0.05
6-P-Glucosamine	25±4	35±7
1-P-GlcNAc	14±2	20±6
UDP-GlcNAc	35±8	40±7
6-P-fructose	0.25±0.02	0.4±0.1
glucose	0.4±0.05	0.5±0.05

Figure S6. GlmS-related replenishment can rescue MA-D1-induced MRSA inhibition and death. The MIC and MBC values of MA-D1 against MRSA with or without 1 mg/mL 6-P-Glucosamine, 1-P-GlcNAc, UDP-GlcNAc, 6-P-fructose or glucose, respectively. Values are the means ± SD.

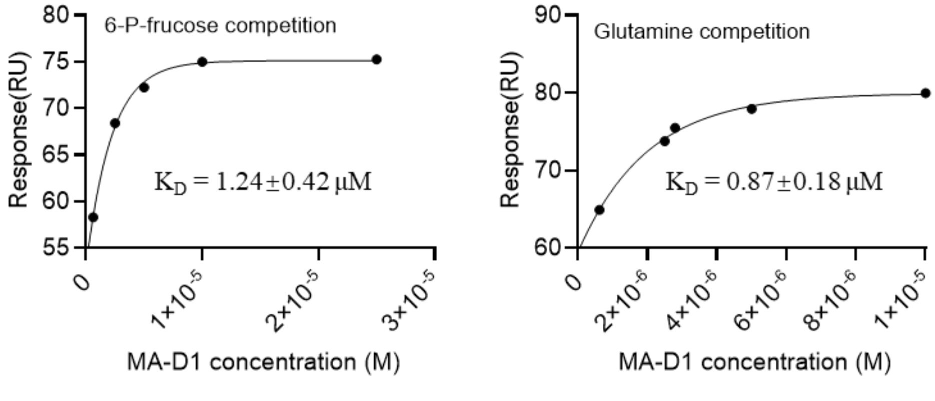

Figure S7. The binding affinity between MA-D1 and GlmS protein under the competition from fructose 6-phosphate or glutamine. GlmS protein was captured on the sensor chip CM5, and the gradient MA-D1 was injected into the SPR system with or without 1 mM fructose 6-phosphate or glutamine.

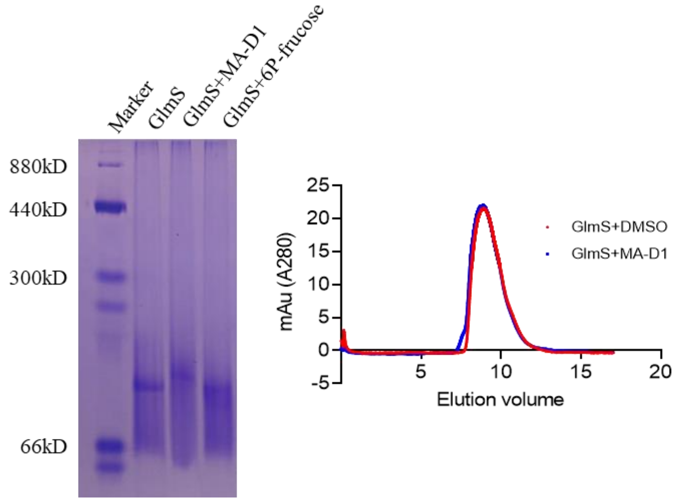

Figure S8. The oligomeric form detection of GlmS protein. (**A**) Native PAGE. (**B**) Size exclusion chromatography.

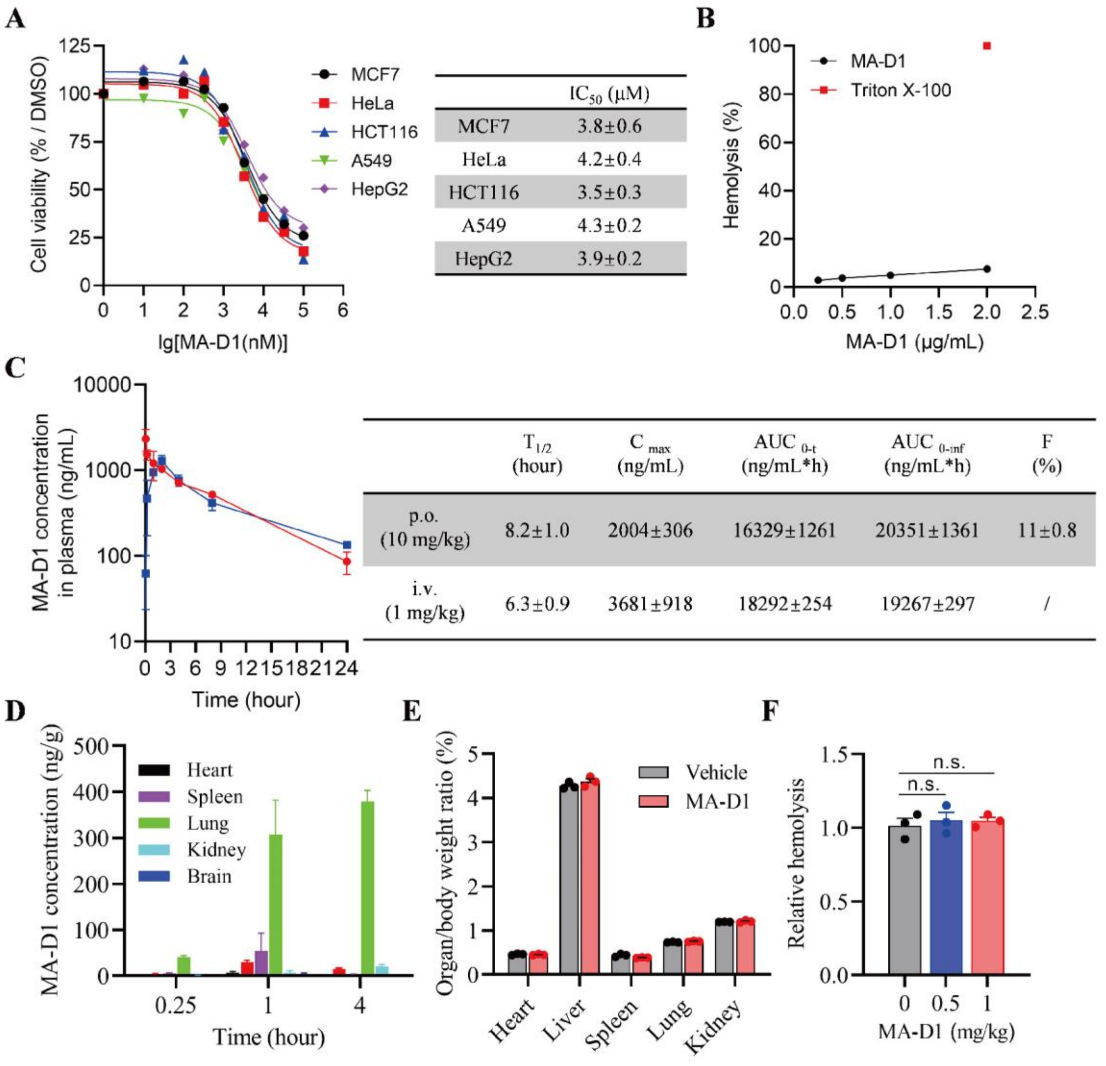

	IC_{50} (μM)
MCF7	3.8±0.6
HeLa	4.2±0.4
HCT116	3.5±0.3
A549	4.3±0.2
HepG2	3.9±0.2

	$T_{1/2}$ (hour)	C_{max} (ng/mL)	$AUC_{0\text{-}t}$ (ng/mL*h)	$AUC_{0\text{-}inf}$ (ng/mL*h)	F (%)
p.o. (10 mg/kg)	8.2±1.0	2004±306	16329±1261	20351±1361	11±0.8
i.v. (1 mg/kg)	6.3±0.9	3681±918	18292±254	19267±297	/

Figure S9. The toxicity, pharmacokinetic parameters and tissue distribution of MA-D1. (A) Cytotoxicity of MA-D1 towards mammalian cells (n=3). (**B**) Normalized hemolysis of mouse

erythrocytes by MA-D1 relative to Triton X-100. (**C**) The pharmacokinetic parameters of MA-D1 in mice (n=3). $T_{1/2}$, half-life; C_{max}, maximum plasma concentration; AUC, area under the curve; F, oral bioavailability. F (%) = [$AUC_{0\text{-}inf}$ (p.o.) * 10] / [$AUC_{0\text{-}inf}$ (i.v.) * 1] * 100%. **(D)** The tissue distribution of MA-D1 in mice (i.v. 1mg/kg, n=3). **(E)** The organ/body weight ratio of mice treated with single MA-D1 (1 mg/kg, i.p) for 24 hours (n=3). (**F**) Systemic administration of MA-D1 (0.5 and 1 mg/kg, i.p.) was hypotoxic based on the hemolytic phenotype in mice (n=3). Values are the means ± SEM. n.s, no significance.

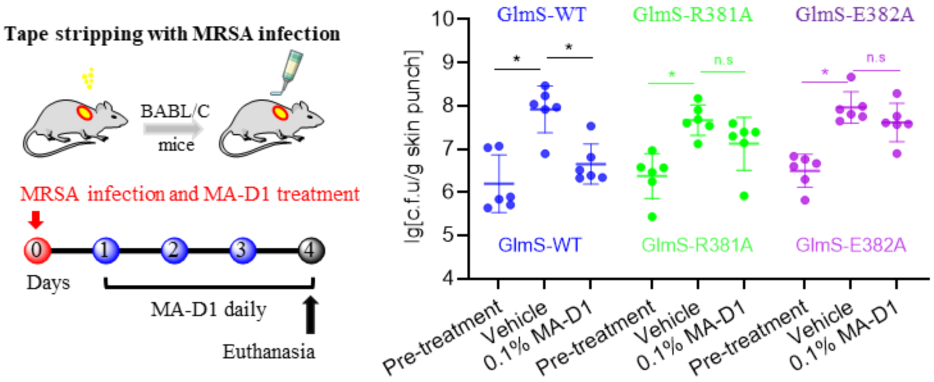

Figure S10. The efficacy of MA-D1 on alleviating infection with MRSA transfected with inactive GlmS mutants in mice (n=6). The designed experimental pattern flow chart (left), and the c.f.u. for surface-attached bacteria (right). Values are the means ± SEM. n.s, no significance, *P<0.05.

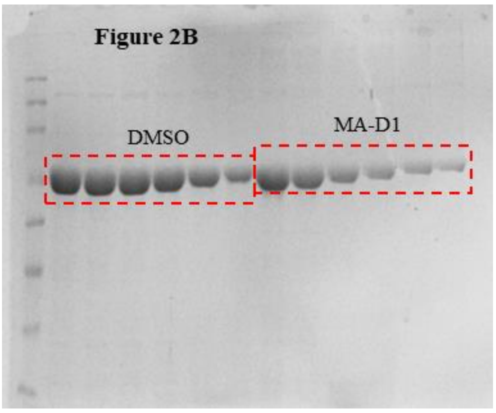

Figure S11. Full scan of western blots.

III) Materials and methods of biology experiments

Antibiotic inhibitory determination

Frozen storage MRSA (-80°C) is inoculated in an antibiotic-free TSB medium. After the overnight cultivation at 37°C, a monoclonal colony is selected and cultured overnight at 37°C in 1 mL of TSB-free medium. Compounds in different work concentrations are added to the 24-well plate with 500 μL of MH medium. After the bacterial solution grows to OD_{600} = 1.0, the bacterial solution is diluted 10^4 times, 10 μL diluents are added on the solid medium plates and incubated overnight at 37°C. For the survival rescue experiments, 1 mM of glucose, fructose 6-phosphate, glucosamine 6-phosphate, and UDP-GlcNAc were added to the TSB medium.

Gel-based protein profiling

MRSA is collected by centrifugation at 4000 rpm for two minutes and then resuspended with PBS buffer. MRSA cells were treated with probes alone to determine and label efficacy and the suitable working concentration of probes (0-150 μM). MRSA cells were pre-treated with gradient native MA-D1 (0-600 μM) for 1 hour, followed by probe 1 or probe 2 (150 μM) treatment for another 2 hours. The treated MRSA was transferred into plates on the ice and illuminated with 365 nm ultraviolet light for 15 minutes; the control group was placed on ice in a dark box. Then, the lysozyme (0.05 mg/mL) was added to every sample and incubated at 37°C for 30 minutes, followed by the centrifugation at 8000 rpm, 4°C, and the supernatant collection. Trichloroacetic acid was added to the supernatant and stood for 30 minutes at -20°C; the supernatant was removed after the centrifugation at 15000 rpm, 4°C for 20 minutes. The precipitate was washed with cold acetone and then centrifuged at 15000 rpm, 4°C for 20 minutes. PBS buffer containing 0.4% SDS was used to dissolve the proteins. The dissolved protein concentration was measured and diluted to 1 mg/mL with PBS buffer. For the gel-based profiling test, AF488-azide (200 μM), VcNa (200 μM), CuSO4 (10 μM), and DTTAA (10 μM) were added to the diluted protein solution and incubated for 1 hour at room temperature to trigger the click reaction. Then, the protein loading buffer was added and analyzed by SDS-PAGE on a Fujifilm LAS-3000 fluorescent system.

MS-based profiling of MA-D1-interacting proteins

For the MS-based profiling test, AF488-azide was replaced by biotin-azide (100 μM) and was

added into the diluted protein solution and incubated for 1 hour at room temperature to conduct the click reaction. Then cold acetone was added and incubated for 4 hours at -20℃. The supernatant was removed after the centrifugation at 15000 rpm, 4℃ for 20 minutes, and the precipitate was dissolved by PBS containing 0.4% SDS. Protein concentration was accurately measured again and diluted to 1 mg/mL with PBS. The avidin beads were washed three times with PBS containing 0.4% SDS, then were added into the diluted protein solution and mixed for 3 hours at room temperature. Next, the mixture was washed three times with PBS containing 0.4% SDS, followed by three times with pure PBS. Resuspending the avidin beads with PBS containing 6M urea, then TCEP (10 mM) was added and incubated for 15 minutes at 65℃. IAA (20 mM) was added and incubated for 30 minutes at room temperature. The supernatant was removed after the centrifugation at 3000 rpm, 4℃ for 3 minutes, and the avidin beads were resuspended with PBS containing 2M urea, followed by the addition of $CaCl_2$ (1 mM) and trypsin (0.5 mg/mL). After overnight digestion at 37℃, pure formic acid collected and acified the supernatant (5% final concentration).

Mass spectrometry

The tryptic peptides based on the above-mentioned method were loaded onto a Dionex C18 Nano Trap Column and, subsequently, were eluted and separated by a Dionex C18 PepMap 100 column. The analysis was conducted by tandem MS followed by high-resolution MS using a coupled Q-Exactive Orbitrap mass spectrometer (Thermo Fisher Scientific) under the guidance of a professional instrument manager. Mobile phases A (2% acetonitrile, adjusted pH to 10.0 using $NH_3 \cdot H_2O$) and B (98% acetonitrile, adjusted pH to 10.0 using $NH_3 \cdot H_2O$) were used. Mobile phase A consisted of 0.1% formic acid in water solution, and mobile phase B consisted of 0.1% formic acid in acetonitrile solution. According to the hydrophobicity of each fraction, an adjusted linear gradient was applied with a flow rate of 350 nL/min. The MS conditions are as follows: under the positive-ion mode, full-scan mass spectra were acquired over the m/z range from 400 to 1800 using the Orbitrap mass analyzer with a resolution of 70000. MS/MS fragmentation was performed in the data-dependent mode, in which the 18 most intense ions were selected from each full-scan mass spectrum for fragmentation by high-energy collision-induced dissociation (HCD). MS/MS spectra were acquired with a resolution of 17500 using the Orbitrap analyzer. Some other parameters in the centroid format: isolation window, 2.0 m/z units; default charge, 2^+; normalized collision energy,

28%; maximum IT, 50 ms; dynamic exclusion, 20s.

Protein identification and quantification

The mass spectrometry data were searched with the SEQUEST algorithm against the corresponding database on Proteome Discovery software. The search was limited to only tryptic peptides, two missed cleavage sites, monoisotopic precursor ions and a peptide tolerance of < 10 ppm. Further refinement of the search results was established by filter settings: the Xcorrvs charge state filter was set to Xcorr values of 1.5, 2.0 and 2.5 for charge states +1, +2 and +3, respectively. The number of different peptides has to be ≥2 and the peptide probability filter was set to 0.001. The static modification of cysteine is +57.0125 Da, and the variable oxidation of methionine is +15.9949 Da.

Protein expression and purification

GlmS, GuaB, AldA and cysteine synthase genes were respectively cloned into the prokaryotic expression vector pET28a(+)-6His-TEV and transfected into BL21 (DE3) *E.coli.* The *E.coli* carrying these plasmids were cultured at 37°C to OD_{600} about 1.0, then 1 mM isopropyl 1-thio-b-D-galactopyranoside was added to induce GlmS protein expression at 18°C for 20 hours. Finally, cells were collected through centrifugation and lysed by sonication in 30 mM Tris-HCl, 200 mM NaCl, pH 8.0 lysis buffer. The soluble proteins in supernatant were first purified using Ni-NTA column (GE Healthcare). The His-tag was removed by using 1 mg/ml TEV enzyme overnight at 4°C, and then cleaned by size exclusion (Superdex 200 10/300 GL column, GE Healthcare) chromatography. The quantity of purified protein was confirmed through SDS-PAGE.

Enzymatic activity test

The enzyme activities of GlmS were detected using LC-MS to derive glucosamine 6-phosphate. Gradient concentrations of MA-D1 were added into PBS buffer containing GlmS protein (10μg/mL) and incubated for 12 hours. Then glutamine and fructose 6-phosphate were added and incubated for another 20 minutes at 37°C. Finally, boric acid (pH 9.0) and NHS-Fmoc in acetonitrile solution were added to the mixture and incubated for 3 hours. UPLC-MS detected the derivatization rate, and the relative GlmS enzymatic activity under different treatments was quantified.

Thermal shift assay

1 mg/mL diluted GlmS protein was pre-incubated with 15 μM MA-D1 or vehicle control within 20 μL final volume for 1 hour at room temperature. The mixture samples were heated at 48-66°C for 5 minutes, followed by centrifugation at 15000 rpm for 10 minutes, and analyzed by SDS-PAGE with coomassie blue staining.

SPR assay

The SPR binding assay was conducted at 25°C using the Biacore T200 SPR system (GE Healthcare). Purified tag-free GlmS protein was captured on the Sensor Chip CM5 (carboxymethylated dextran surface) using an Amine Coupling kit. After fixing the GlmS protein on the chip, gradient concentrations of MA-D1 diluents with or without 1 mM glutamine or fructose 6-phosphate were sequentially injected. All experiments were conducted in PBS-P running buffer (Cytiva). The binding affinities (Kd) were calculated by fitting binding affinity and kinetics and analyzed using Biacore T200 Evaluation software (version 2.0, GE Healthcare).

Transmission electron microscope

MA-D1 (4μg/mL) or vehicle was added to the medium for 3 hours. Then, MRSA was collected by centrifugation and suspended with 2.5% glutaraldehyde fixing solution at 4°C for 2 hours. After fixation, it was washed three times with PBS buffer. Then, MRSA was embedded into liquid paraffin and fixed at 4°C overnight. It was washed three times with PBS buffer again and stored at 4°C before transmission electron microscopy (JEOL JEM-1400 Flash, HC) detection.

Molecular docking

The GlmS/MA-D1 molecular docking was performed based on the reported structure of GlmS from *S. aureus* (PDB ID, 4S1W). One monomer structure of the GlmS isomerase domain was isolated, and redundant components were removed with MOE software. MA-D1 was docked into the pocket of the GlmS isomerase domain with a standard routine. Finally, Pymol was used to output the pictures in the Figures.

Resistance development assay

The resistance frequency of MRSA to antibiotics was evaluated in a sequential generation manner. First, the MIC of MA-D1, linezolid, vancomycin, and teicoplanin to MRSA treatment was determined, as described previously. Next, each sub-MIC concentration of antibiotics was re-diluted to 2×10^5 c.f.u/mL and used for the next-generation MIC determination. The procedure mentioned above was repeated for 14 passages.

Hemolysis assay

Different concentrations of MA-D1 were added into 100 uL 6% mice erythrocyte solution, incubated at 37°C for 30 min, and centrifuged at 2500 rpm for 10 min, then absorbance of the supernatant (OD_{405}) was measured using a Envision microplate reader (Perkin Elmer, USA). Triton X-100 was used as a positive control.

Cytotoxicity measurement

Cytotoxicity was measured by CellTiter-Glo reagent (Promega, USA) according to the manufacturer's instructions. Briefly, after the MA-D1 treatment, cells in a 96-well plate were added with CellTiter-Glo reagent for 10 min incubation at room temperature, then the chemiluminescence was recorded by Envision plate reader (PerkinElmer, USA) as the readout of cell viability.

RNA isolation and RT-PCR

Total RNA isolation (Cat# R6934, OMEGA Bio-Tek, GA), reverse transcription (Cat# 11141ES10, Yeasen, China), and real-time RT-PCR using CFX^{96} (Biorad) with SYBR mixture (Cat# 11201es08, Yeasen, China) were performed according to the manufacturer's standard instructions. 16SrRNA-F, GAACCGCATGGTTCAAAAGT; 16SrRNA-R, TATGCATCGTTGCCTTGGTA. SigB-F, AGTGTACATGTTCCGAGACG; SigB-R, GTCCCATTTCCATTGCTTC.

Mice treatment

All the SPF animals were bought from the company (Charles River, China) and acclimated for a week before treatment in the laboratory animal center. All involved animal experiments were conducted in the barrier facility of the Laboratory Animal Center, Sinoresearch (Beijing) Biotechnology Co., Ltd, and approved by the Institutional Animal Use and Care Committee (approved number: ZYZC2024070001J).

Acute toxicity of MA-D1 in mice. Eight weeks female BABL/C mice were intraperitoneally treated with MA-D1 (1 mg/kg) or vehicle (negative control, 5% DMSO, 20% solution HS-50, 75% water). After 24 hours, the heart, liver, spleen, lung and kidney were collected and weighted., and the organ weight/body weight ratios were calculated.

The pharmacokinetics of MA-D1 was tested by a single intravenous (i.v. 1 mg/kg) or oral administration (p.o. 10 mg/kg) in 8-week-old female BABL/C mice (n = 3). Blood samples were collected at 0.083, 0.25, 0.5, 1, 2, 4, 8, and 24 h post-administration through orbital venous plexus by capillary, EDTA-K_2 anticoagulant. LC-MS/MS quantified the MA-D1 concentration in plasma samples, and pharmacokinetic parameter analysis was conducted using PKsolver 2.0.

Tissue distribution parameters of MA-D1 were tested by a single intravenous of MA-D1 (i.v. 1 mg/kg) in 8-week-old female BABL/C mice (n = 3). The heart, liver, spleen, lung, kidney and brain were collected at 0.25, 1, and 4 h post-administration, then the MA-D1 concentration in different tissues were determined by LC-MS/MS.

Skin infection animal models. 6-8 weeks female BABL/C mice were shaved and anesthetized, then seven times tape stripping on the back skin was implemented, and following MRSA ($2x10^6$ c.f.u in 20 uL PBS/mouse) was applied on the skin. After 24 hours of infection, grouped randomly and vehicle, MA (0.1%, 1%, 10% in vehicle) or linezolid (10% in the vehicle) were topical application at infection site once daily for the next four days. Adult SD rats were shaved and anesthetized, and then ~ two centimeters of back skin was surgically removed; after hemostasis and disinfection, MRSE (1.5×10^8 c.f.u/rat) infection in the incision site was performed once daily for the next four days to induce severe infection. Grouped randomly and administrated with vehicle, 1% or 10% MA at infection site once daily for three days. 6 hours after the final application, animals were euthanized, the skin or scabs were detached and weighted, cut up the removed skin and homogenized by vortex in sterile PBS. Then, c.f.u of fractions were determined by serial dilutions, following spotted on the agar culture plate at 37°C overnight, counting the spots and calculating them. The same skin area of the different mice was collected and fixed in 10% formalin, embedded in paraffin, all skin sections sectioned at a thickness of 5 μm, then hematoxylin and eosin (H&E) were performed following the standard operation, microscopic examination, and photographs were taken under a regular light microscope.

Systemic infection model: eight weeks old female BABL/C mice were rendered neutropenic by administering cyclophosphamide intraperitoneally (100 mg/kg, 3 days, then mice were infected intraperitoneally with MRSA (1-2x10^8 c.f.u/mouse), and were randomly assigned to treatment groups (n=10). Mice were administered intraperitoneally with MA-D1 (0.5 or 1 mg/kg, 10 mice) or vehicle (negative control) at time points 1, 24 and 48 h post-infection, and were monitored for survival for seven days following infection.

Hemolytic phenotype test of MA-D1 *in vivo*: eight weeks old female BABL/C mice were infected intraperitoneally with single MA-D1 (0.5 or 1 mg/kg, 3 mice) or vehicle. Fresh blood of mice was collected after 24 hours and centrifuged for 10 min, then the absorbance (OD_{405}) of serum was measured using a Envision microplate reader (Perkin Elmer, USA).

Quantification and statistical analysis.

Values in this study were expressed as mean ± standard error of the mean (SEM). For comparisons with only two groups with the same standard deviation, the student's t-test was used. Analysis of variance (ANOVA) with Tukey's post-test was used to compare values among different experimental groups (one-way ANOVA for comparisons between groups; two-way ANOVA for comparisons of changes between different groups from different interventions. Numbers per group were mentioned in the Figure legends. $P < 0.05$ was considered significant.

IV) Synthesis of probes

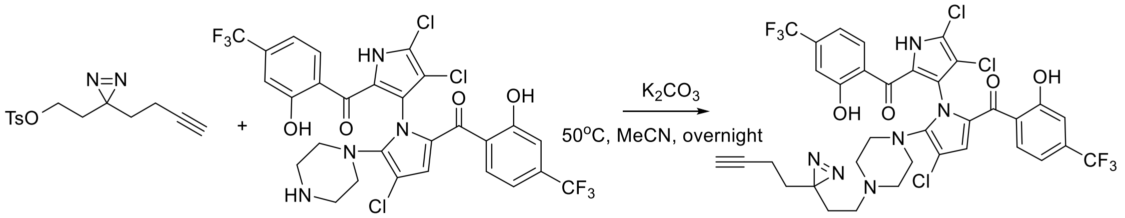

S1 **MA1** **Probe 1**

The solution of compound **S1**[1] (1.61 g, 5.5 mmol), **MA1**[2] (3.2 g, 4.6 mmol) and K_2CO_3 (3.17 g, 22.9 mmol) in MeCN (100 mL) was stirred at 50 °C for overnight. After stirring, the reaction mixture was quenched with water and extracted by EA (3×100 mL). The organic phase was dried by Na_2SO_4, evaporated in vacuo, and purified by silica gel column and preparative LC to obtain **probe 1** (2.15 g, 47.9%).

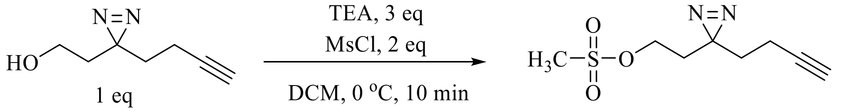

S2 **S3**

Compound **S2** (2.30 g, 16.7 mmol) was dissolved in dry DCM (300 mL). The mixture was stirred at 0 °C for 10 min, and TEA (9.26 mL) was added, followed by dropwise addition of MsCl (2.58 mL). The mixture was stirred at RT for 8 h. TLC showed that the reaction was completed. The reaction was quenched with water. The mixture was extracted with DCM (3 × 100 mL). The combined organic layers were dried with anhydrous sodium sulfate and concentrated under vacuum. The crude material was purified by column chromatography (PE: EA = 20:1) to afford compound **S3**.

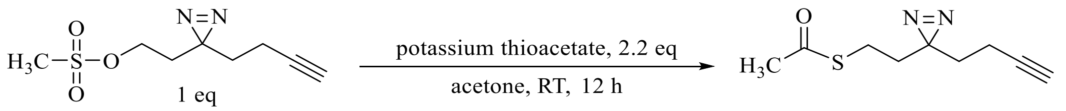

S3 **S4**

Compound **S3** (2.76 g, 128 mmol) and potassium thioacetate (3.21 g, 281 mmol) were dissolved in acetone (500 mL) under argon. The mixture was stirred at RT for 12 h. TLC showed that the reaction was completed. Water was added to dissolve the reaction mixture. The mixture was extracted with DCM (2 × 100 mL). The combined organic layers were dried with anhydrous sodium

sulfate and concentrated under vacuum. The crude material was purified by column chromatography (PE: DCM = 40:1) to afford compound **S4**.

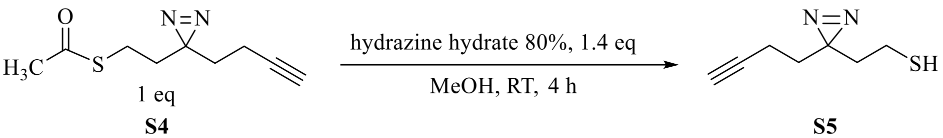

1 eq

S4

S5

Compound **S4** (2.35 g, 12.0 mmol) was dissolved in dry MeOH (100 mL), and hydrazine hydrate solution (80%, 1 mL) was added dropwise at RT. The mixture was stirred at RT for 4 h. TLC showed that the reaction was completed. The reaction was quenched with saturated aqueous $NaHCO_3$ and extracted with EA (3 × 100 mL). The combined organic layers were dried with anhydrous sodium sulfate and concentrated under vacuum. The crude material was purified by column chromatography (PE: EA = 20:1) to afford compound **S5**.

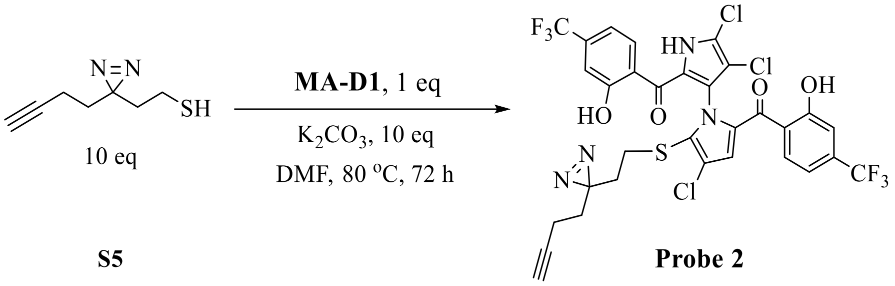

Compound **S5** (1.03 g, 6.7 mmol), **MA-D1** (0.43 g, 0.67 mmol) and anhydrous K_2CO_3 (0.93 g, 6.7 mmol) were dissolved in extra dry DMF (100 mL) under argon. The mixture was stirred at 80 °C for 72 h. The reaction was quenched with water and extracted with EA (3 × 100 mL). The combined organic layers were dried with anhydrous sodium sulfate and concentrated under vacuum. The crude material was purified by column chromatography (PE: EA = 4:1) and by preparative LC to afford the **probe 2**.

V) NMR Spectra

Characterization data: ^{1}H NMR Spectra, ^{13}C NMR Spectra of all compounds

Probe1: ^{1}**H NMR** (600 MHz, Chloroform-*d*) δ 8.02 (d, *J* = 8.2 Hz, 1H), 7.66 (d, *J* = 8.3 Hz, 1H), 7.32 (s, 1H), 7.22 (d, *J* = 8.3 Hz, 1H), 7.18 (s, 1H), 6.77 (s, 1H), 6.68 (d, *J* = 8.3 Hz, 1H), 2.86 (s, 2H), 2.41 (s, 2H), 2.17 (d, *J* = 7.5 Hz, 3H), 2.00 (d, *J* = 6.1 Hz, 2H), 1.63 (t, *J* = 7.2 Hz, 2H), 1.55 (q, *J* = 7.8 Hz, 2H). ^{13}**C NMR** (151 MHz, cdcl$_3$) δ 186.03, 185.86, 171.48, 162.37, 161.28, 142.95, 136.97, 110.28, 106.80, 60.62, 21.21, 14.32. **HRMS**: calculated $C_{35}H_{28}Cl_3F_6N_6O_4$, $[M+H]^+$=815.114, measured mass:815.111.

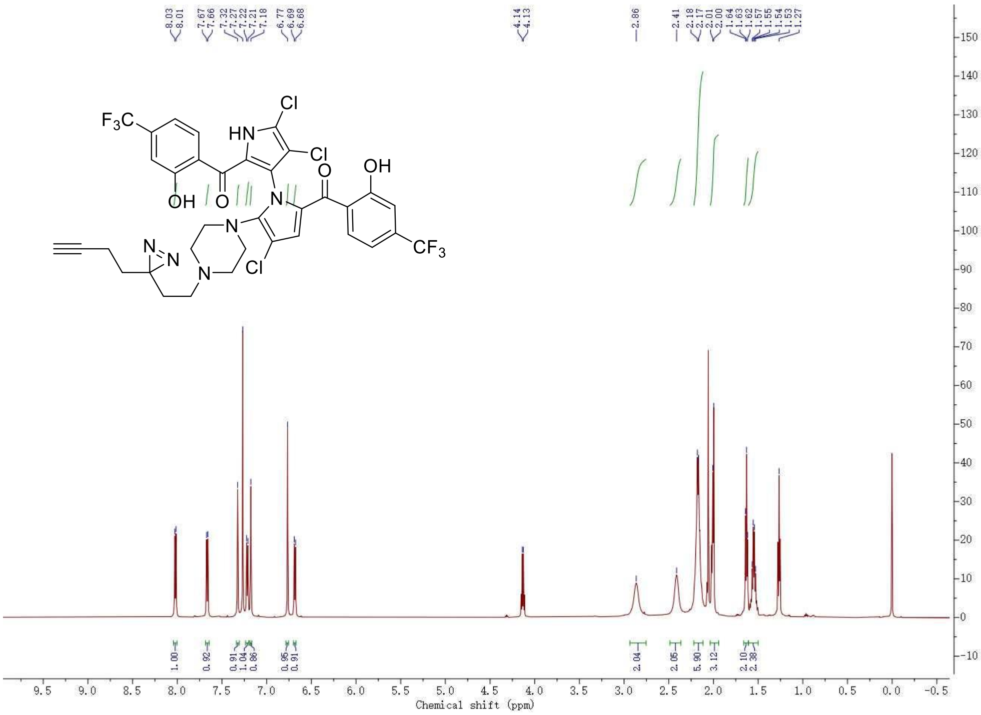

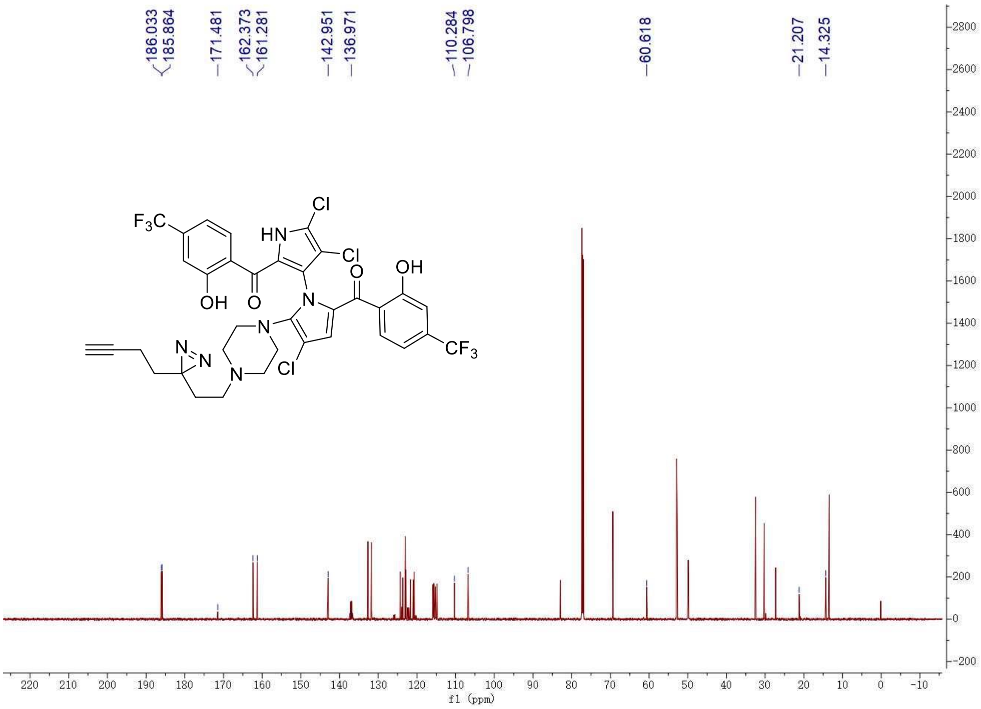

Compound **S3**: **^{1}H-NMR** (600 MHz, $CDCl_3$) δ 4.08 (t, *J* = 6.2 Hz, 2H), 3.06 (s, 3H), 2.06 – 1.99 (m, 3H), 1.90 (t, *J* = 6.2 Hz, 2H), 1.70 (t, *J* = 7.8 Hz, 2H); **^{13}C-NMR** (150 MHz, $CDCl_3$) δ 82.5, 69.6, 64.1, 37.7, 33.0, 32.3, 26.0, 13.3.

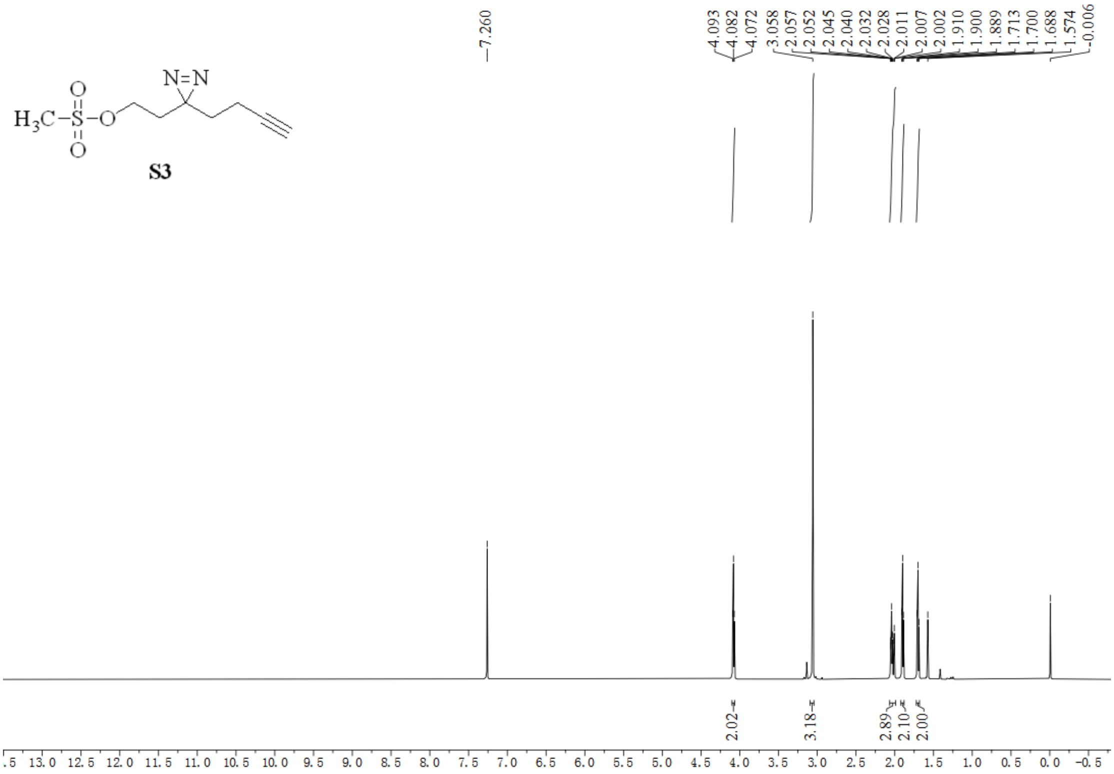

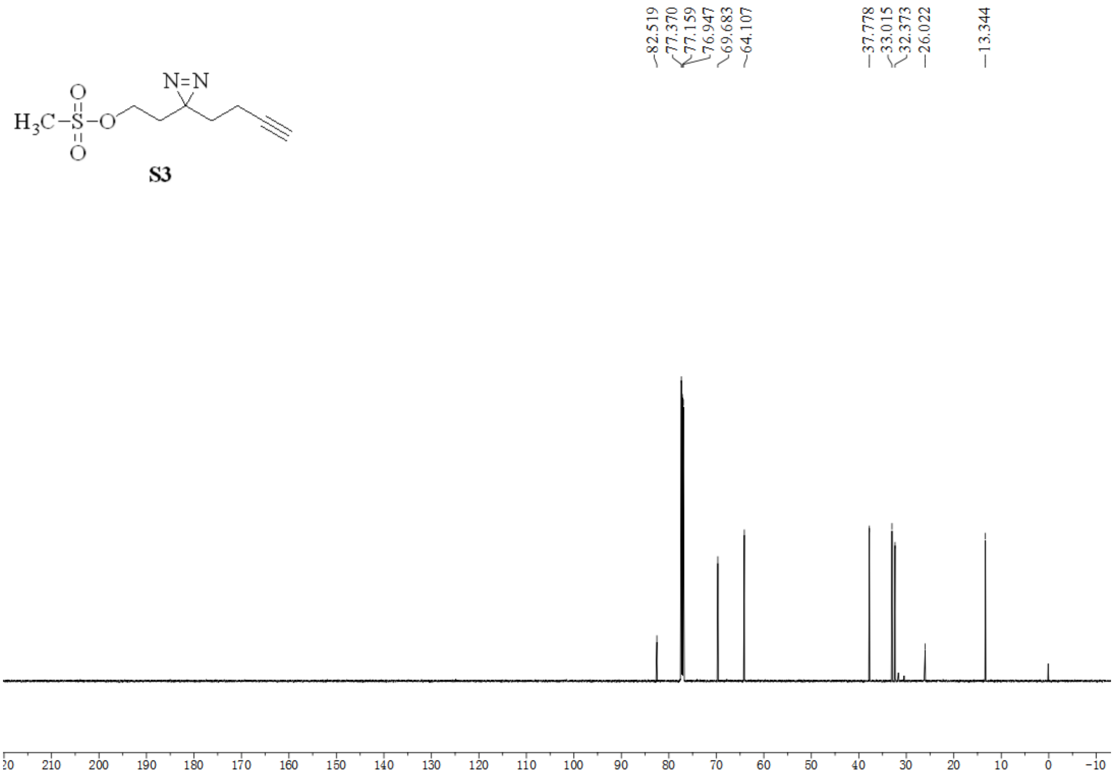

Compound **S4**: **^{1}H-NMR** (600 MHz, $CDCl_3$) δ 2.69 (t, *J* = 7.8 Hz, 2H), 2.32 (s, 3H), 2.06 – 1.97 (m, 3H), 1.73 – 1.64 (m, 4H); **^{13}C-NMR** (150 MHz, $CDCl_3$) δ 195.3, 82.6, 69.4, 33.3, 32.1, 30.7, 27.7, 23.5, 13.4.

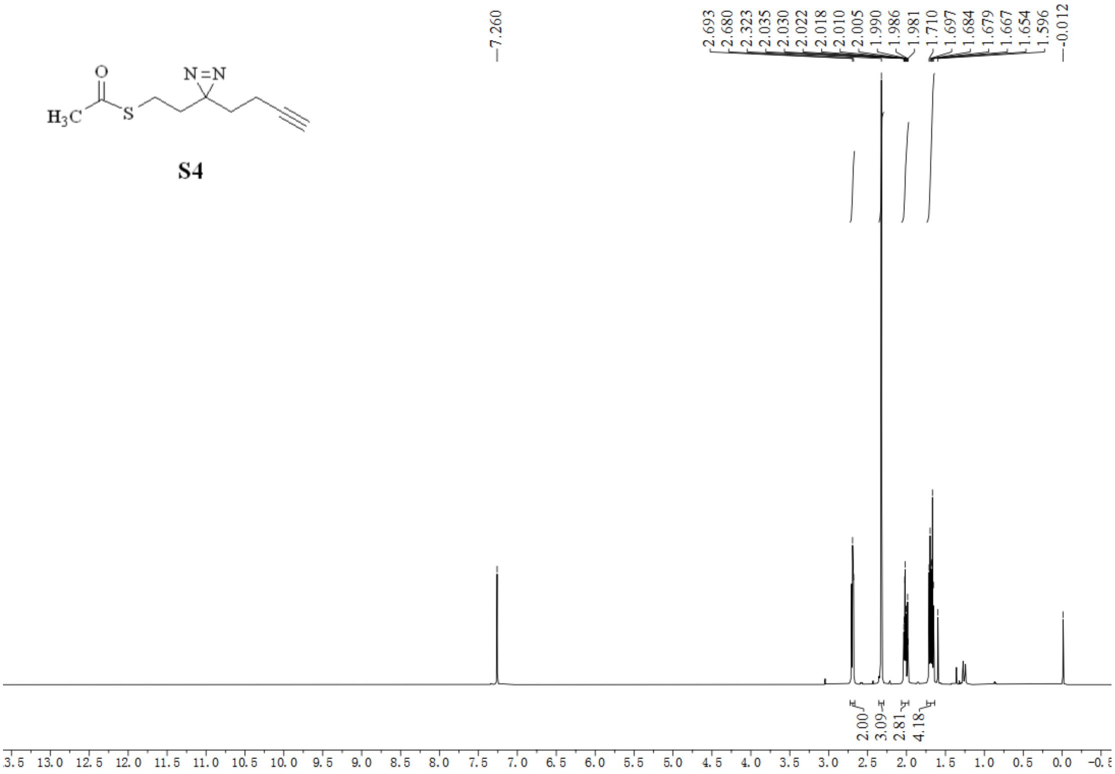

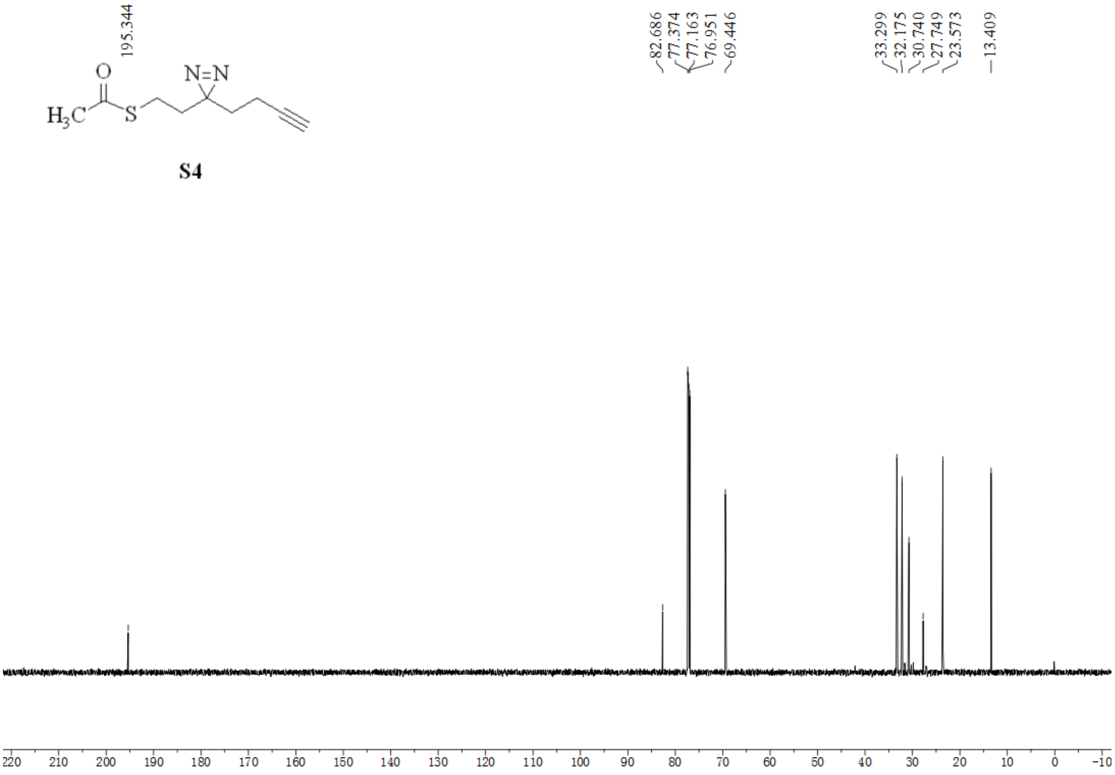

Compound **S5**: **^{1}H-NMR** (400 MHz, $CDCl_3$) δ 2.49 – 2.40 (m, 2H), 2.06 – 1.99 (m, 3H), 1.86 – 1.78 (m, 2H), 1.66 (t, *J* = 7.2 Hz, 2H); **^{13}C-NMR** (100 MHz, $CDCl_3$) δ 82.6, 69.5, 32.7, 32.4, 32.3, 27.6, 13.4.

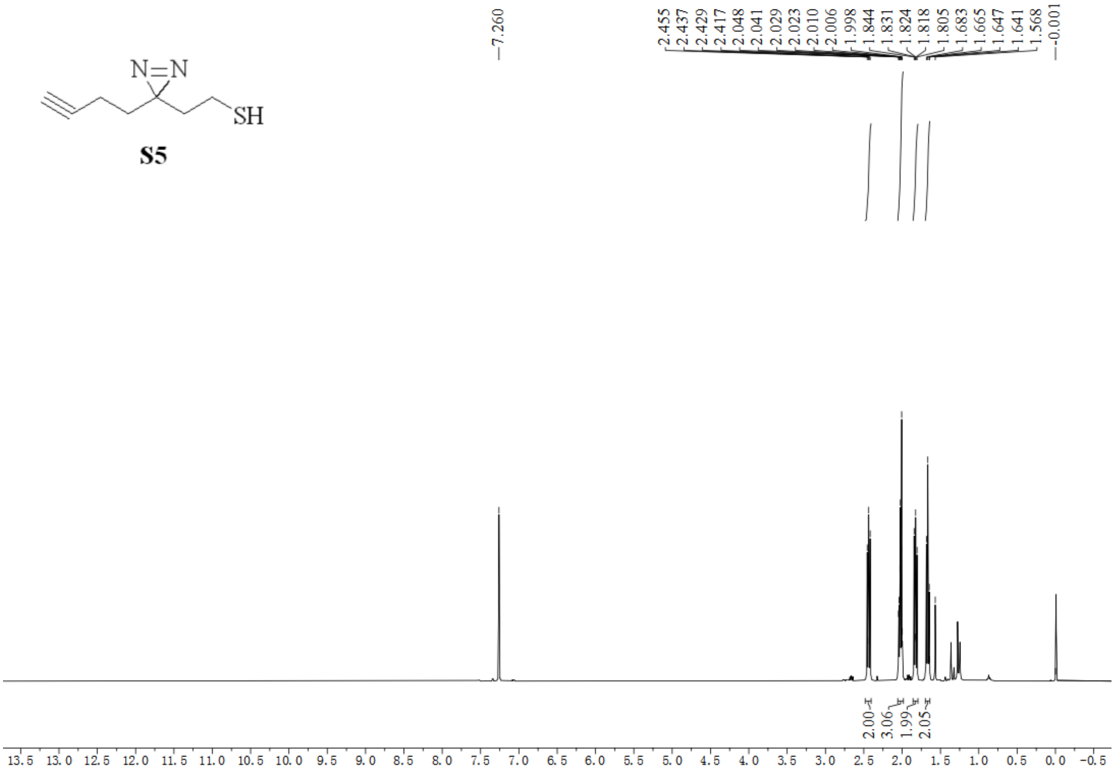

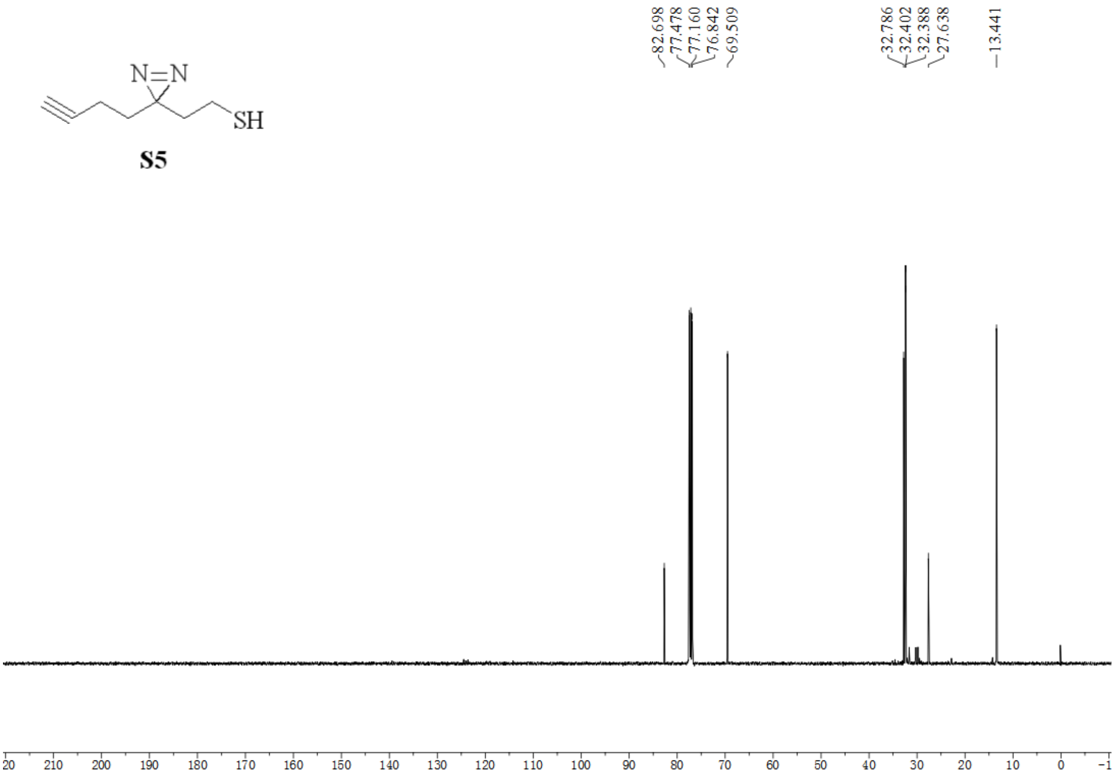

Probe 2: **^{1}H-NMR** (400 MHz, $CDCl_3$) δ 8.01 (d, *J* = 8.4 Hz, 1H), 7.57 (d, *J* = 8.4 Hz, 1H), 7.36 – 7.32 (m, 1H), 7.25 – 7.17 (m, 2H), 6.85 (s, 1H), 6.66 – 6.60 (m, 1H), 2.37 – 2.28 (m, 1H), 2.20 – 2.10 (m, 1H), 2.00 – 1.96 (m, 1H), 1.94 – 1.88 (m, 2H), 1.53 – 1.32 (m, 4H); **^{13}C-NMR** (100 MHz, $CDCl_3$) δ 187.1, 185.0, 162.8, 160.6, 137.6 (q, *J* = 33 Hz), 136.8 (q, *J* = 33 Hz), 132.9, 130.9, 130.9, 130.5, 124.6, 124.1, 124.1, 123.1 (q, *J* = 272 Hz), 123.0 (q, *J* = 272 Hz), 122.7, 121.3, 121.1, 120.9, 116.1, 115.7, 115.2, 115.0, 110.2, 82.5, 69.5, 33.0, 32.0, 32.0, 27.2, 13.3. **HRMS** (ESI) calculated for $C_{31}H_{18}Cl_3F_6N_4O_4S$ $[M-H]^-$: 761.0024, found 761.0024.

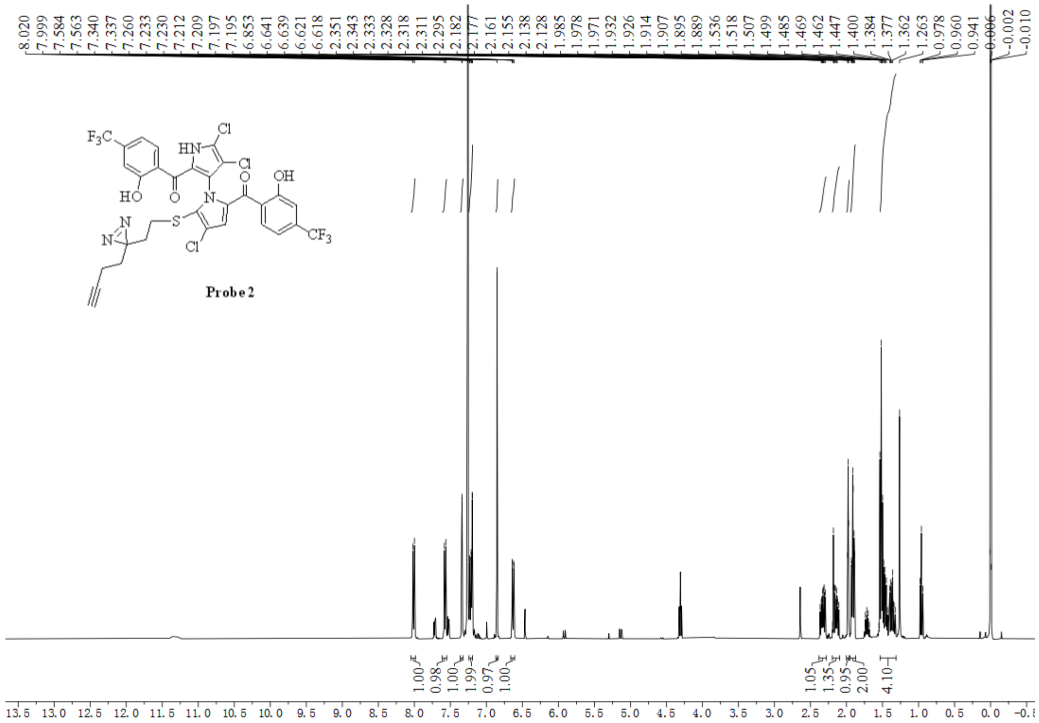

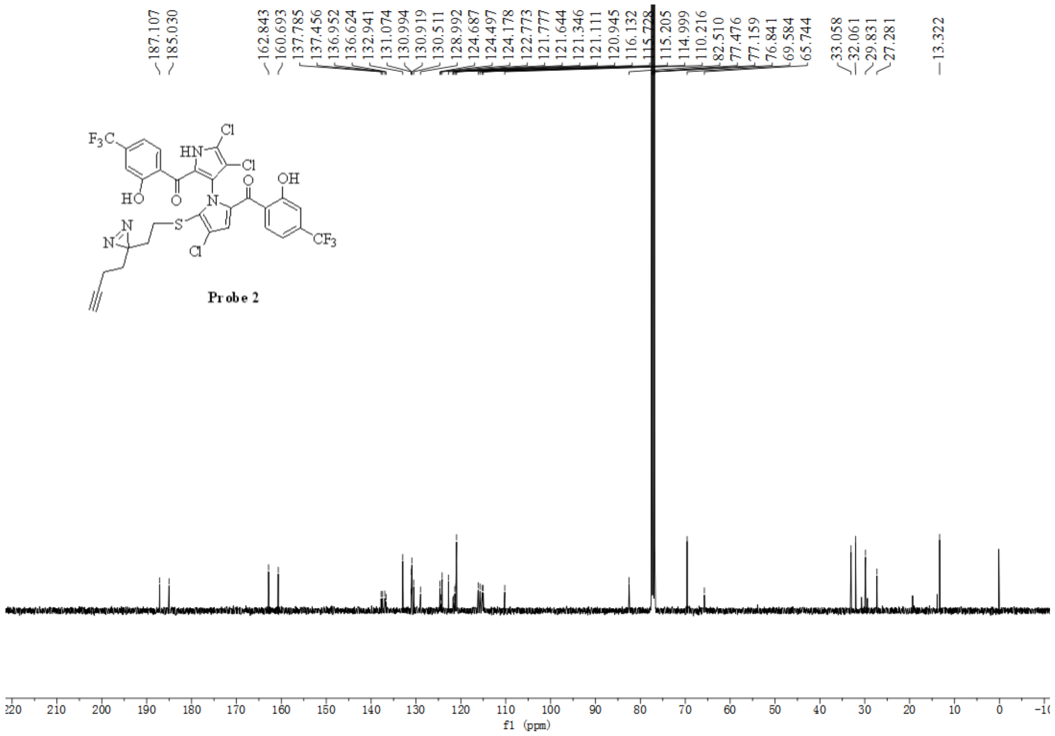

VI) Reference:

[1] He, D. *et al.* Quantitative and Comparative Profiling of Protease Substrates through a Genetically Encoded Multifunctional Photocrosslinker. *Angew. Chem. Int. Ed. Engl.* **56**, 14521-14525, (2017).

[2] Stepek, I. A. *et al.* Antibiotic Discovery with Synthetic Fermentation: Library Assembly, Phenotypic Screening, and Mechanism of Action of beta-Peptides Targeting Penicillin-Binding Proteins. *ACS. Chem. Biol.* **14**, 1030-1040, (2019).
